# Expression of a Truncated ATHB17 Protein in Maize Increases Ear Weight at Silking

**DOI:** 10.1371/journal.pone.0094238

**Published:** 2014-04-15

**Authors:** Elena A. Rice, Abha Khandelwal, Robert A. Creelman, Cara Griffith, Jeffrey E. Ahrens, J. Philip Taylor, Lesley R. Murphy, Siva Manjunath, Rebecca L. Thompson, Matthew J. Lingard, Stephanie L. Back, Huachun Larue, Bonnie R. Brayton, Amanda J. Burek, Shiv Tiwari, Luc Adam, James A. Morrell, Rico A. Caldo, Qing Huai, Jean-Louis K. Kouadio, Rosemarie Kuehn, Anagha M. Sant, William J. Wingbermuehle, Rodrigo Sala, Matt Foster, Josh D. Kinser, Radha Mohanty, Dongming Jiang, Todd E. Ziegler, Mingya G. Huang, Saritha V. Kuriakose, Kyle Skottke, Peter P. Repetti, T. Lynne Reuber, Thomas G. Ruff, Marie E. Petracek, Paul J. Loida

**Affiliations:** 1 Monsanto Company, St. Louis, Missouri, United States of America; 2 Mendel Biotechnology Inc., Hayward, California, United States of America; 3 Dupont-Pioneer Hi-Bred International, Waipahu, Hawaii, United States of America; 4 Dupont-Pioneer Hi-Bred International, Hayward, California, United States of America; 5 ABCAM, Burlingame, California, United States of America; 6 Monsanto Company, Cambridge, Massachusetts, United States of America; 7 Monsanto Company, Bangalore, Karnataka, India; University of Massachusetts Amherst, United States of America

## Abstract

*ATHB17* (AT2G01430) is an Arabidopsis gene encoding a member of the α-subclass of the homeodomain leucine zipper class II (HD-Zip II) family of transcription factors. The ATHB17 monomer contains four domains common to all class II HD-Zip proteins: a putative repression domain adjacent to a homeodomain, leucine zipper, and carboxy terminal domain. However, it also possesses a unique N-terminus not present in other members of the family. In this study we demonstrate that the unique 73 amino acid N-terminus is involved in regulation of cellular localization of ATHB17. The ATHB17 protein is shown to function as a transcriptional repressor and an EAR-like motif is identified within the putative repression domain of ATHB17. Transformation of maize with an ATHB17 expression construct leads to the expression of ATHB17Δ113, a truncated protein lacking the first 113 amino acids which encodes a significant portion of the repression domain. Because ATHB17Δ113 lacks the repression domain, the protein cannot directly affect the transcription of its target genes. ATHB17Δ113 can homodimerize, form heterodimers with maize endogenous HD-Zip II proteins, and bind to target DNA sequences; thus, ATHB17Δ113 may interfere with HD-Zip II mediated transcriptional activity via a dominant negative mechanism. We provide evidence that maize HD-Zip II proteins function as transcriptional repressors and that ATHB17Δ113 relieves this HD-Zip II mediated transcriptional repression activity. Expression of ATHB17Δ113 in maize leads to increased ear size at silking and, therefore, may enhance sink potential. We hypothesize that this phenotype could be a result of modulation of endogenous HD-Zip II pathways in maize.

## Introduction

HD-Zip proteins are a family of plant transcription factors that are found broadly across plant species [1] and that play an important role in regulating plant growth and development. In addition, many HD-Zip proteins are involved in mediating the response of plants to a range of environmental conditions. The family is characterized by the presence of a leucine zipper (LZ) domain adjacent to a homeodomain (HD) in the C-terminus [2], [3] and comprises four distinct subfamilies designated as I, II, III, and IV [1]. The sub-families, or classes, were defined based on the sequences of the conserved HD and LZ domains, gene structures, and the presence of additional class-specific motifs and domains. HD-Zip proteins bind DNA as homo- or heterodimers and their specificity of DNA recognition may depend on a precise spacing between the DNA-binding and dimerization regions [4]. Many HD-Zip proteins were shown to function as active repressors of gene expression [4], [5], [6], [7] and also to down regulate transcription of genes within the HD-Zip family [6], [8].

Although the functions of many HD-Zip family members are poorly understood and their downstream targets are mainly unknown, recent studies have demonstrated a significant progression toward the elucidation of potential functions of HD-Zip proteins. The evidence indicates that these transcription factors play an important role in regulation of plant growth and development. HD-Zip IV proteins are expressed in the outer cell layers of various plant organs and are involved in the regulation of apical meristem and shoot organ development [9], [10], [11], [12], [13]. Members of the HD-Zip III family function in the maintenance and developmental regulation of apical mersteim and lateral organ polarity, most likely, through regulation of auxin transport [14], [15], [16], [17], [18], [19]. Members of the HD-Zip I family have been associated with plant growth response to abiotic stresses [20], [21], [22]. The role of HD-Zip II proteins is still mainly undiscovered, as the null mutations in many HD-Zip II genes produce no detectable phenotypes [8], [23], [24]. Suggested functions for these proteins are based on studies of their expression patterns under different conditions. ATHB2 was one of the first HD-Zip II genes described and shown to be regulated by the ratio of red to far red light (R/FR)[13]. ATHB4 is involved in the shade avoidance response [8]. Two other HD-Zip II proteins, HAT1 and HAT3 negatively regulate cell proliferation during leaf development under high R/FR light [25]. Additionally, HAT1 is also involved in reproductive development of Arabidopsis [26]. Additional functions of HAT3 and ATHB4 were elucidated using double mutations that led to simultaneous loss of both proteins, and it was demonstrated that they are involved in the regulation of cell proliferation during leaf development [27] and, potentially, regulation of shoot apical meristem activity and embryo development [28].

Systematic analysis by Zhao et al. [22] identified 18 HD-Zip IIs out of 55 HD-Zip genes in the maize genome, but functional characterization of the maize HD-Zip IIs has not been reported. While phylogenetic analysis suggests that HD-Zip II genes are closely related among monocot species, there are few ortholog pairs between monocot and dicot species [20], [22]. Genes closely related to *ATHB17* have been identified in rice and maize, OsHox3 and Zmhdz18, respectively [22]. ATHB17 is expressed in the root quiescent center of Arabidopsis [24], [29], while OsHox3 is expressed in the panicle after flowering [20]. OsHox3 is reported to function as transcriptional repressors [30]. No physiological functions have been attributed to OsHox3 or Zmhdz18 proteins and very little is known about the potential functions of ATHB17 protein. No phenotypes were observed in a knoc-kout mutant of ATHB17 by Hymus et al. [24]. In another study, seedlings of an Arabidopsis knock-out mutant showed ABA-insensitive and drought-sensitive phenotypes, but only during a short post-germinating period, indicating ATHB17 might function in a narrow window of plant development [31]. Constitutive over-expression of ATHB17 in Arabidopsis conferred several phenotypes including altered leaf development, changes in leaf morphology, plastid number and size, and increased photosynthetic capacity [24].

Here we demonstrate that ATHB17 protein functions as a transcriptional repressor in Arabidopsis and corn protoplasts and contains a newly identified EAR-like motif responsible for repression activity. The protein is localized to both the cytoplasm and nuclei and its unique N-terminus could be responsible for regulation of protein localization. Similar to other members of HD-Zip II class, ATHB17 forms homodimers and heterodimers within its own class and dimerization is required for high affinity DNA binding and repression activity. Transformation of the *ATHB17* gene into maize under the control of a monocot promoter results in the expression of a truncated protein that functions as a dominant-negative regulator that can modify activity of endogenous maize HD-Zip II transcription factors. Additionally, we demonstrate that maize HD-Zip II proteins function as repressors of gene expression and are expressed in a tissue- and development-specific manner. A greater number of HD-Zip II genes are transcribed in reproductive tissues, especially the ear inflorescence, than in any other tissues analyzed. Expression of ATHB17Δ113 protein in maize leads to changes in ear growth resulting in increased ear size at early reproductive stages and potentially increased sink size, which could be a result of ATHB17Δ113 modulating the activity of the endogenous maize HD-Zip II proteins.

## Results and Discussion

### ATHB17 is a Transcriptional Repressor Containing EAR-like Motif

**Figure 1:**
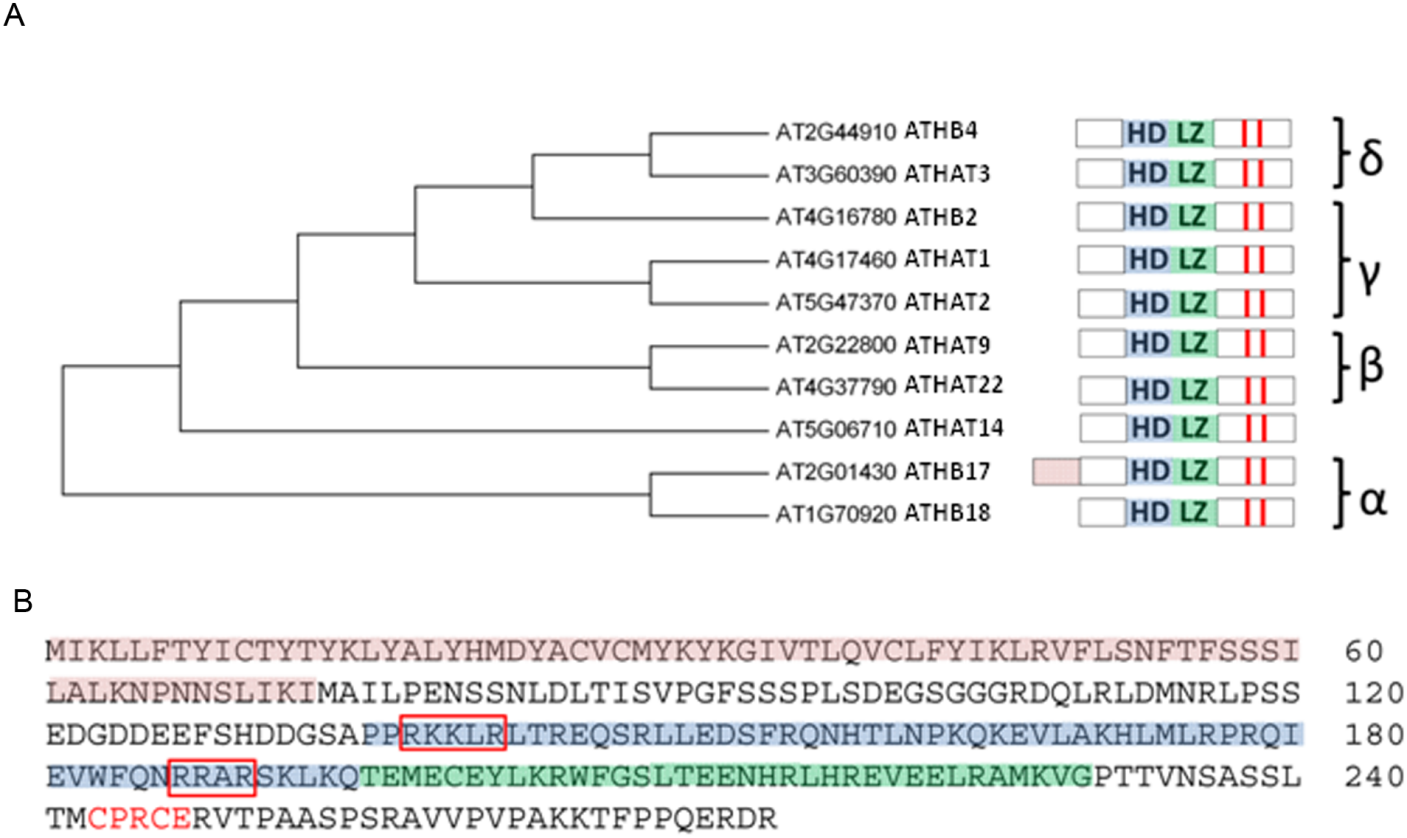
ATHB17 is a member of the α subclass within the HD-Zip II protein family. (A) represents the dendrogram and the domain architecture of the ATHB17 homologs. ATHB17 contains a typical homeodomain (HD; blue shading) and a leucine zipper motif (LZ; green shading) adjacent to the C-terminus of the HD. Red bars indicate conserved cysteines in the C-terminus. (B) shows the protein sequence of ATHB17. ATHB17 contains a unique N-terminal extension (red shading) rich in cysteines and tyrosines. Additional structural feature identified for ATHB17 is a nuclear localization signal (red boxes). Downstream of the LZ motif is a putative redox sensing motif (CPXCE; red letters).

*ATHB17* encodes a protein consisting of 275 amino acids (Figure 1) and contains five domains, four of which are typical for HD-Zip II proteins. Common to all HD-Zip II members is the presence of a homeodomain (HD) with an immediately adjacent leucine zipper (LZ), a hypothetical N-terminal repression domain, and a redox sensing motif (CPXCE), characteristic to all HD-Zip II proteins downstream of the leucine zipper motif [1], [25]. In addition, ATHB17 has a unique 73 amino acid N-terminal extension rich in tyrosine and cysteine that has not been identified in any other HD-Zip II proteins (Figure 1).

**Figure 2:**
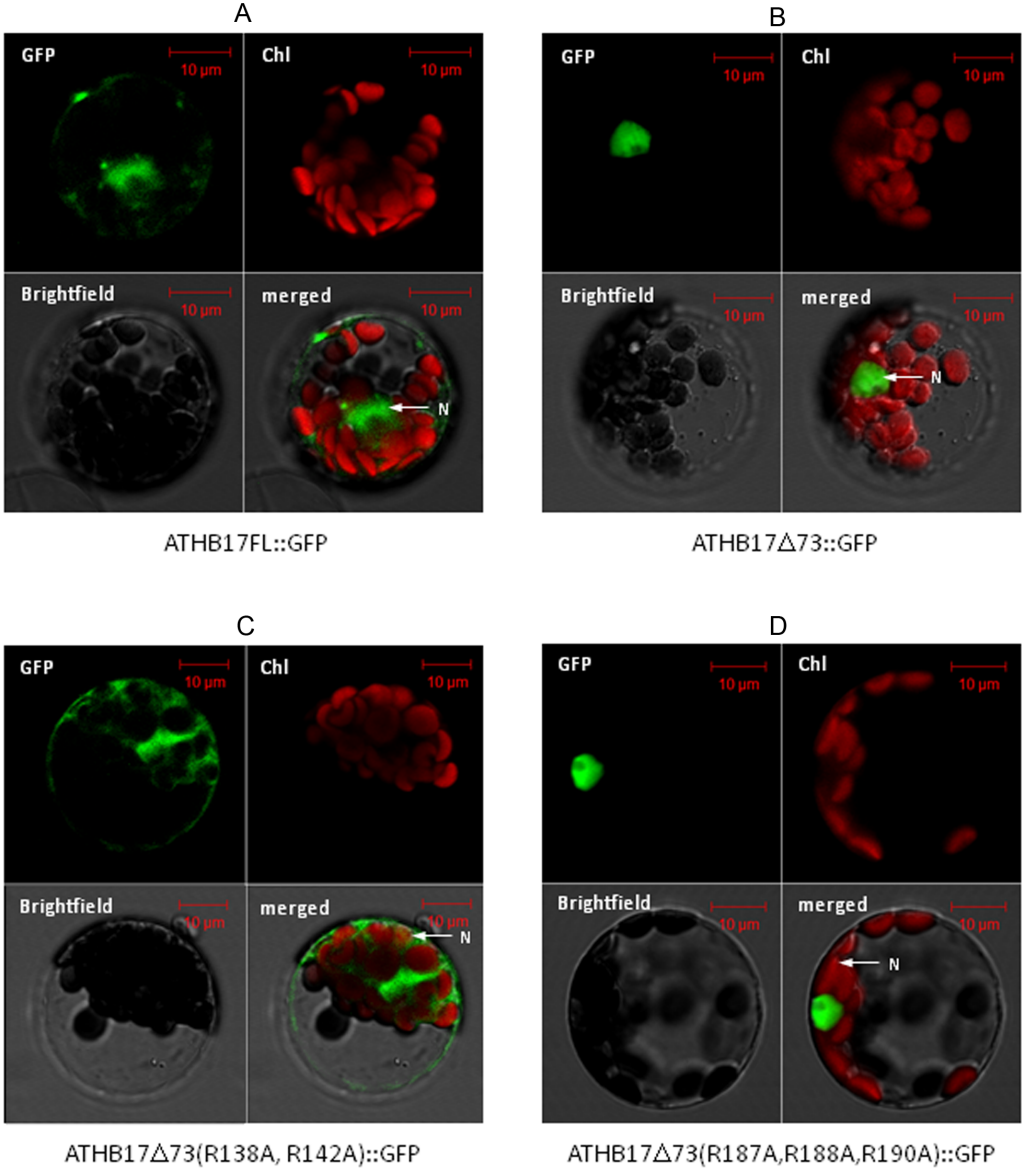
Representative fluorescence micrographs of Arabidopsis mesophyll protoplasts. Purified protoplasts were transformed with (A) full length ATHB17:GFP or (B) a variant lacking the first 73 amino acids (ATHB17Δ73:GFP). The full length ATHB17:GFP fusion protein localizes to the nucleus and cytosol (A) in Arabidopsis protoplasts and ATHB17Δ73:GFP is localized exclusively in the nucleus when the unique N- terminus is removed (B). Two regions with a cluster of basic (K and R) residues were identified as putative nuclear localization signals. The arginine residues were mutated to alanines in these regions in the Δ73 variant to disrupt translocation. (C) R138A and R142A mutations in ATHB17 Δ73 disrupted nuclear retention while (D) R187A, R188A and R190A mutations did not alter the nuclear localization of the Δ73 variant.

To understand the potential functions of ATHB17 protein, we attempted to characterize the unique N-terminal extension and determine if the protein has the ability activate or repress transcription. Based on bioinformatic analysis using the TransMem+ (GCG) program, the unique N-terminus of ATHB17 may possess at least one, and possibly two, transmembrane domains at amino acids 4 to 23 and 35 to 57 or amino acids 4 to 23 and 40 to 62, respectively. The presence of these putative transmembrane domains suggests that ATHB17 could be tethered to a membrane [32] and translocated to the nucleus as a result of a specific regulatory mechanism [33]. Numerous examples exist of transcription factors that are sequestered in the cytoplasm, then translocated to the nucleus following a signaling event [32], [34], [35]. To determine if the N-terminus of ATHB17 may play a role in mediating cytoplasmic or nuclear localization, C-terminal green fluorescent protein (GFP) fusions of ATHB17 and a deletion variant lacking the unique N- terminus (ATHB17Δ73) were transformed into Arabidopsis protoplasts and the subcellular localization of each fusion protein was determined (Figures 2A and 2B). Deletion of amino acid residues 1 to 73, which contain the putative transmembrane domains, eliminated the cytoplasmic localization of ATHB17 with only nuclear localization apparent. Two putative nuclear localization signals (NLS) were identified at amino acids 136 to 142 and amino acids 187 to 194 using the localization prediction tool pSort [36]. The putative NLSs were recognized as highly basic regions that is a common characteristic of NLS [37]. Arginines within each putative NLS were mutated to alanine and C-terminal GFP fusions of these variants were transformed into protoplasts. Only one variant (ATHB17Δ73 R138A R142A) caused a loss of nuclear localization (Figure 2C). Mutations of arginine to alanine at positions 187, 188, and 190 did not alter the nuclear localization of the Δ73 variant (Figure 2D). Therefore, the motif at amino acids 136 to 142 is the sole NLS necessary for targeting the ATHB17 protein into the nucleus.

**Figure 3:**
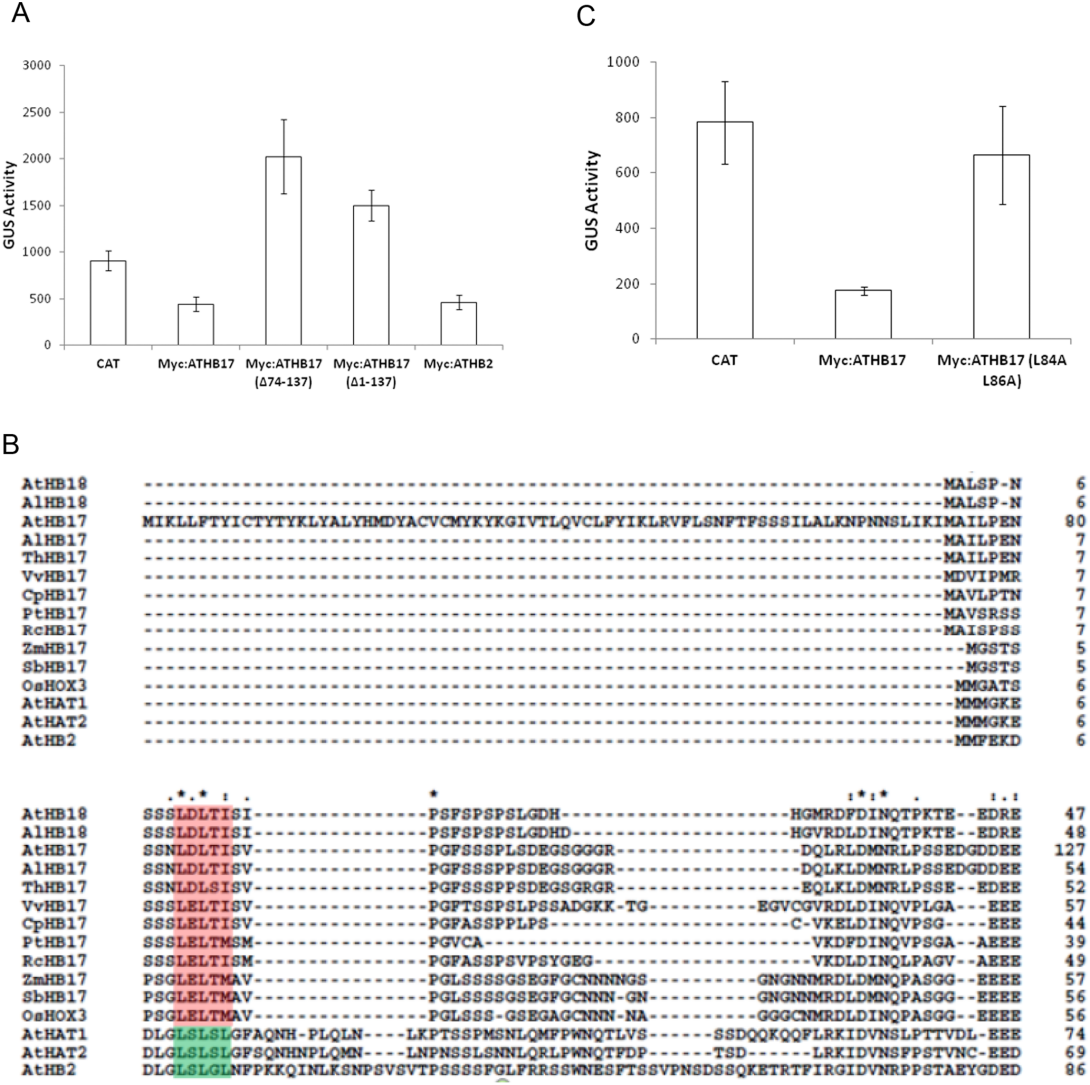
Effect of various mutations on the transcriptional repression and identification of EAR-like motif. (A) Arabidopsis mesophyll protoplasts were co-transformed with the *pHAT1*::*GUS* reporter gene and the following effector constructs: chloramphenicol acetyltransferase (CAT), ATHB17, ATHB17 with amino acids 74–137 deleted, ATHB17 with amino acids 1–137 deleted and ATHB2. Data are mean fluorescence readings measuring GUS-mediated substrate (4-methylumbelliferyl-beta-D-glucuronide) conversion. Error bars represent ± SD of three replicates. (B) Multiple alignment of the protein sequences similar to the amino terminus of ATHB17 from publically available HD-Zip II α subfamily proteins. Gaps are indicated by dashes. A putative EAR motif is shaded in red for the α-subclass, while the EAR motif identified in [25] is shaded in green. Abbreviations for species are as follows: At *Arabidopsis thaliana*; Al *Arabidopsis lyrata*; Th *Thellungiella halophila*; Vv *Vitis vinifera*; Cp *Carica papaya*; Pt *Populus trichocarpa*; Rc *Ricinus communis*; Zm *Zea mays*; Sb *Sorghum bicolor*; Os *Oryza sativa*. (C) *ATHB17*, *ATHB17* with the EAR motif mutated (ATHB17 L84A L86A), or *CAT* effector constructs were co-transformed into Arabidopsis mesophyll protoplasts with the *pHAT1*::*GUS* reporter gene. Data are mean fluorescence readings measuring GUS-mediated substrate (4-methylumbelliferyl-beta-D-glucuronide) conversion. Error bars represent ± SD of three replicates.

To determine if an active transcriptional repression domain is present in ATHB17, various deletions were constructed and tested in an Arabidopsis protoplast-based transcriptional activation/repression assay. The *pHAT1*::*GUS* reporter construct was used because the basal level of expression allowed for observation of both activation and repression. Protoplast transformation with *35S*::*chloramphenicol acetyltransferase* (*CAT*) was used as a positive control because CAT is not expected to bind to DNA. Consequently, the amount of expression observed using CAT should reflect the basal level of reporter gene expression. Transformation of genes encoding proteins that bind to the promoter and cause activation or repression is expected to alter the basal reporter gene expression [38]. Results with specific effector constructs may vary between individual experiments from slight differences in transformation efficiency [38]. Transformation with ATHB2 was chosen as a positive control as it has been previously characterized as a transcriptional repressor [5], [6], [39]. Strong repression was observed when full-length ATHB17 or full length ATHB2 was co-transformed with the *pHAT1*::*GUS* reporter (Figure 3A). Expression of the *pHAT1*::*GUS* reporter was not repressed by ATHB17 variants lacking amino acids 1–137 (*ATHB17* Δ*1–137*) or 74–137 (*ATHB17* Δ*74–137)* (Figure 3A), suggesting that all or part of a repression domain is present between amino acids 74 and 137. The increased expression observed in the presence of truncated ATHB17 proteins would be expected if these truncated proteins compete for binding to the promoter with transcriptional repressors that are endogenously expressed in the protoplasts.

The repression activity of several HD-Zip II proteins is associated with the presence of an ERF-associated amphiphilic repression (EAR) motif (LxLxL) [25], a well-described transcriptional repression motif [40], [41]. Two class II HD-Zips (ATHB2 and ATHAT2) also have been functionally characterized as repressors using *in vitro* or *in vivo* assays [5], [6], [39]. However, an EAR motif was not identified in ATHB4, ATHAT14, ATHB17, or ATHB18 proteins [25]. To characterize repression activity observed for ATHB17 protein, we identified orthologous genes from several plant species and compiled sequences available in GenBank, including sequences for ATHB17 and ATHB18. A multiple sequence alignment of the N-terminus of these proteins is shown in Figure 3B. We were able to identify an EAR-like putative motif (LxLxI/M) at amino acids 84 to 86 related to the EAR transcriptional repression motif (LxLxL) [41]. A smaller putative EAR-like motif is located at amino acids 61 to 63. Mutating the conserved leucine residues L84 and L86 to alanine (L84A and L86A) caused a significant loss of repression (Figure 3C), while mutating leucine residues L61 and L63 to alanine (L61A and L63A) did not impact the level of repression (S. Tiwari, data not shown). This suggests that the EAR-like motif at amino acids 84 and 86 is required to produce repression.

### ATHB17 Expression in Maize Results in a Truncated Protein that Lacks the Repression Domain

**Figure 4:**
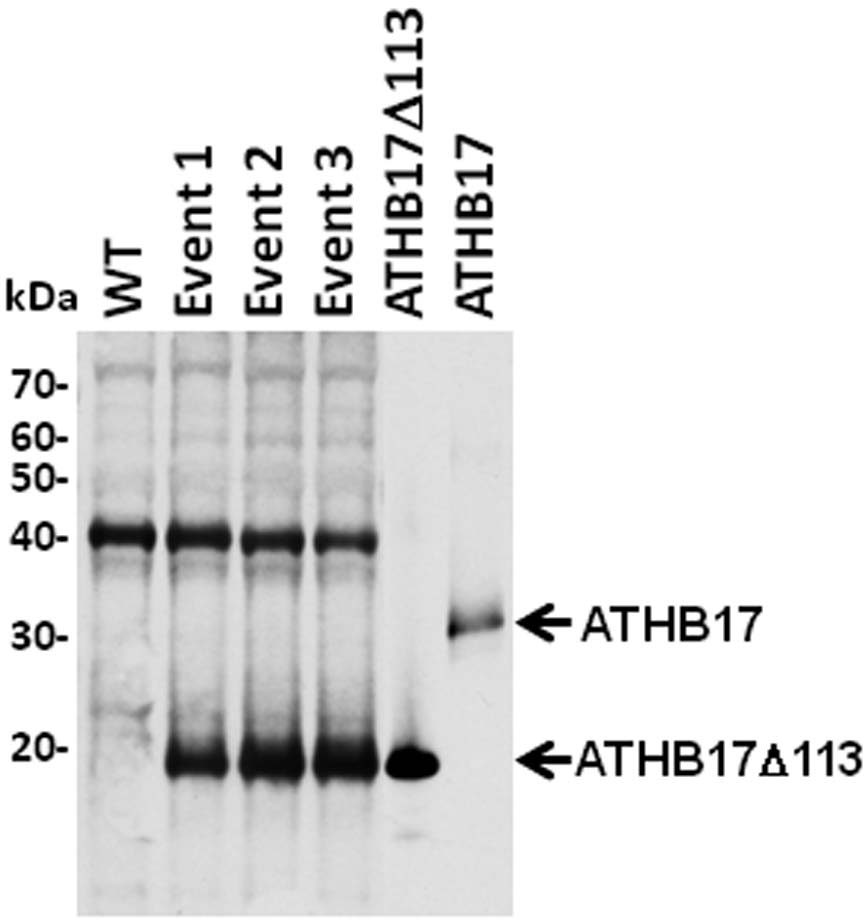
Western blot analysis showing truncated ATHB17 protein in transgenic maize events. Extracts from WT and transgenic maize events were separated on SDS-PAGE, transferred on to nitrocellulose membrane, and probed with ATHB17 antibody. An *E. coli* purified preparation of full-length and truncated protein was also analyzed for comparison with plant expressed protein as a control. Molecular weight markers are shown on the left.

The coding region of the full-length *ATHB17* gene under the control of a constitutive monocot promoter was transformed into maize. No signal corresponding to the full-length protein (expected molecular weight ∼32 kDa) was observed in the transgenic maize plants on the Western blot shown in Figure 4. A band with an apparent molecular weight of approximately 20 kDa was clearly identified in the transgenic maize plants. The ∼20 kDa signal identified in the transgenic plants co-migrated with the signal produced by the truncated ATHB17 protein produced in *E. coli* which was run on the same gel as a standard (Figure 4). As expected, no signal corresponding to the full length or truncated ATHB17 proteins was detected in wild-type plants. A band with the apparent molecular weight of approximately 40 kDa was observed in both wild-type and transgenic events suggesting that the signal is due to the non-specific interaction between the ATHB17 antibody and endogenous maize proteins. Sequence analysis of the *ATHB17* transcript produced in maize demonstrated that, as a result of splicing, a truncated transcript was produced resulting in an ATHB17 protein lacking the first 113 amino acids compared to the sequence of ATHB17 found in Arabidopsis (hereafter referred to as ATHB17Δ113) (Figure S1A–S1C). Mapping of the maize *ATHB17* transcript sequence to the transformation vector revealed that the splicing donor site originates in the intron region of the construct, and that the splicing acceptor site is located within the coding region of the *ATHB17* gene, as shown in Figure S1C. Although in Arabidopsis no splice variants of the *ATHB17* gene were observed, the difference in the recognition of the splice donor and acceptor sites between monocots and dicots is well described [42]. The truncated ATHB17 protein identified by Western blot analysis corresponds to the size of the protein predicted to be translated from the transcript sequence (Figure S1B).

As a result of the loss of the first 113 amino acid residues, the protein expressed in maize lacks its unique N-terminus containing putative transmembrane domains and a large part of the repression domain including the ERF-associated Amphiphilic Repression (EAR)-like motif, but has intact HD and LZ domains (Figure S1D). Because ATHB17Δ113 retains dimerization and DNA binding domains but lacks the repression domain,we hypothesized that the truncated protein may affect the functions of the endogenous HD-Zip II proteins via a dominant-negative mechanism. To examine this hypothesis, we evaluated the properties of ATHB17Δ113 and the phenotypes associated with the expression of the truncated protein in maize.

### ATHB17Δ113 Protein Localizes to the Nucleus in Maize, Binds to Consensus DNA Sequences, and Forms Homodimers and Heterodimers with Maize HD-Zip II Proteins

**Figure 5:**
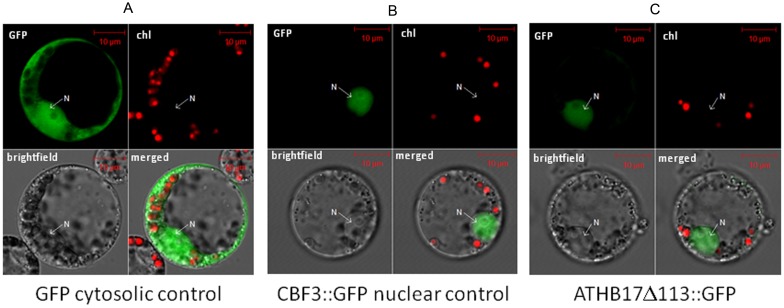
ATHB17Δ113 is localized to nucleus. Fluorescence micrographs of maize leaf protoplasts transformed with (A) GFP alone, (B) AtCBF3 fused to GFP as positive control for nuclear localization or (C) ATHB17Δ113 fused with GFP. Protoplasts from each construct were analyzed by laser scanning microscopy for GFP and chlorophyll fluorescence and acquired images were merged on bright-field image using the Image examiner (See Materials and Methods for details).

To evaluate the localization of ATHB17Δ113 in maize protoplasts, we transformed C-terminal GFP fusions of ATHB17Δ113 into maize protoplasts. A signal was observed only in nuclei, which suggests that, *in planta,* all expressed protein is transported into the nucleus (Figure 5). ATHB17Δ113 would not be expected to be tethered to the membrane because of the absence of the putative transmembrane domains present at amino acids 4 to 23 and 35 to 57 or amino acids 4 to 23 and 40 to 62. To understand if the DNA-binding properties of ATHB17Δ113 have changed as a result of the truncation, we examined the DNA-binding properties of the ATHB17Δ113 *in vitro* using a surface plasmon resonance (SPR) assay. HD-Zip I and II proteins bind to similar *cis* elements (CAAT(N)ATTG) under *in vitro* conditions [4], [30], [43], [44]. HD-Zip II members bind preferentially to the pseudo-palindromic sequence CAAT(C/G)ATTG [4], whereas members of the HD-Zip I subfamily bind preferentially to a pseudo-palindromic sequence that differs from that of HD-Zip II at the central nucleotide (CAAT(A/T)ATTG) [1]. As shown in Table 1, ATHB17Δ113 binds efficiently to both DNA targets with the equilibrium dissociation constant (K_D_) of 37.7±14 nM and 20.4±3.3 nM for class I and class II DNA, respectively. The slightly higher affinity for class II DNA remained consistent throughout our kinetic analyses, underscoring the class II DNA recognition preference by ATHB17Δ113. To determine if truncation of the N-terminus leads to changes in the specific sequence requirements for DNA binding, we examined the effects of mutating several nucleotides in the pseudo-palindromic sequence (Table S1). These data confirmed that ATHB17Δ113 specifically recognizes the pseudo-palindromic consensus sequence (CAAT(N)ATTG), consistent with previous characterization of other HD-Zip II proteins [4]. Additionally, we mutated amino acids within the HD (V182Q185 N186) that should make specific base pair contacts with DNA [45]. As expected, mutating the residues corresponding to V182, Q185, and N186 (*ATHB17 V182A Q185A N186A)* completely abolished DNA binding (Table 1).

**Table 1:** ATHB17Δ113 can bind both Class II and Class I DNA targets and ATHB17Δ113 containing V182A-Q185A-N186A mutation cannot bind Class II DNA target in *in vitro* assay (measured by Surface Plasmon Resonance (SPR).

Protein	Sequence	k*_on_*(1/Ms)×10^5^	k*_off_*(1/s)×10^−4^	K*_D_*(nM)	Relative K*_D_*
ATHB17Δ113	CAGA*CAAT* ***C*** *ATTG*CGGC (Class II)	42.3±16.0	880.6±416	20.4±3.3	1.0
ATHB17Δ113	CAGA*CAAT* ***T*** *ATTG*CGGC (Class I)	29.0±16.4	1012.7±387	37.7±14.0	1.8
ATHB17Δ113	CAG*CTCAGTCTGA*GGC (non-consensus)	-	-	NB	-
ATHB17Δ113 (V182A-Q185A-N186A)	CAGA*CAAT* ***C*** *ATTG*CGGC (Class II)	-	-	NB	-

Binding affinities and Kinetic constants of ATHB17Δ113 interacting with Class I and Class II type DNA, measured by Biacore 2000, globally fitted. SPR measurements with Biacore 2000 were at 25°C in HBS-EP, 100 ug/ml BSA (10 mM HEPES pH 7.4, 150 mM NaCl, 3 mM EDTA, 0.005% Tween-20,100 ug/ml BSA). Equilibrium dissociation constant K_D_ = k_off_/k_on._

**Figure 6:**
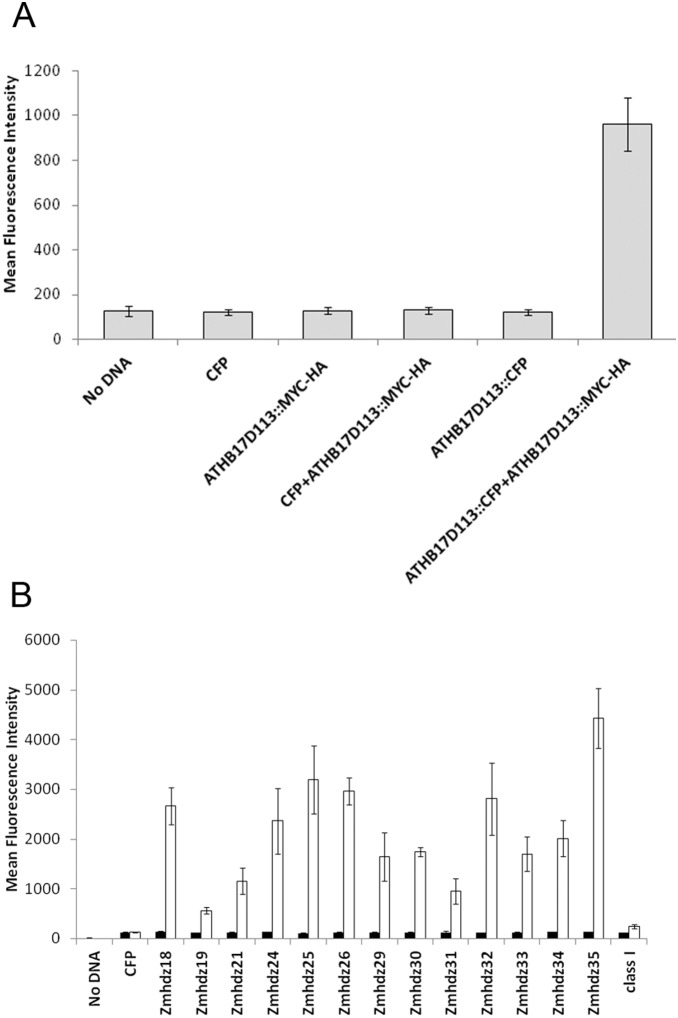
ATHB17Δ113 forms homodimers and makes heterodimers with maize HD-Zip II proteins. Maize leaf protoplasts were (A) mock- transformed or transformed with constructs expressing CFP, MYC::HA and CFP tagged ATHB17Δ113 alone or CFP and ATHB17Δ113::CFP in combination with ATHB17Δ113::MYC construct, (B) transformed (filled bars) with constructs expressing CFP or each of the CFP tagged HD-Zip IIs alone or co-transformed (empty bars) with the construct expressing ATHB17Δ113::MYC-HA. Cellular extracts were co-immunoprecipitated using anti-MYC antibody and the immunoprecipitated complex was probed with anti-CFP antibody and visualized using phycoerythrin-conjugated reporter molecules. One HD-Zip I, Zmhdz3, was used to evaluate interaction with ATHB17Δ113. Nomenclature of HD-Zip II and HD-Zip I constructs is based on Zhao et al.[22].

The ability of ATHB17Δ113 to form homodimers was evaluated in maize protoplasts using a bead-based immunoprecipitation assay with Luminex on-bead detection. A C-terminal MYC-HA fusion of ATHB17Δ113 was co-transformed into maize leaf protoplasts with a C-terminal CFP fusion of ATHB17Δ113. The protein complexes were precipitated with anti-MYC or anti CFP magnetic beads and the expression of the protein of interest was confirmed with biotinylated antibody against HA or CFP (data not shown). Proteins in the dimer were precipitated with anti MYC magnetic beads and detected with biotinylated antibody against CFP. Transformation of either ATHB17Δ113::MYC-HA or ATHB17Δ113::CFP alone did not lead to a signal above background level. However, co-transformation of ATHB17Δ113::MYC-HA with ATHB17Δ113::CFP did lead to a signal (Figure 6A), demonstrating that ATHB17Δ113 can homodimerize.

HD-Zip proteins have been shown to interact with members of their own family, but not with other families of HD-Zip proteins [4]. To identify maize proteins that potentially interact with ATHB17, we performed a yeast two-hybrid screen of a maize cDNA library using ATHB17Δ113 as a bait. We identified several maize HD-Zip II proteins, but none of the other classes of HD-Zip proteins, as putative interactors with ATHB17Δ113 (data not shown). The results of the yeast two-hybrid screen indicate that ATHB17Δ113 may specifically interact with other HD-Zip II proteins when expressed in maize. To confirm this, we selected 13 of the 18 HD-Zip II proteins as representative of the sub-classes in maize and tested them for interaction with ATHB17Δ113 in maize protoplasts using the bead-based co-immunoprecipitation assay.

The C-terminal MYC-HA fusion of ATHB17Δ113 was co-transformed into maize leaf protoplasts with a C-terminal CFP fusion of maize HD-Zip II coding sequences (Figure 6B). The proteins were exposed to antibody against MYC coupled to the magnetic beads and then precipitated. Antibody against CFP conjugated to biotin was used to determine if the complex included maize HD-Zip II proteins. As shown in Figure 3A, no signal above background level was identified when either ATHB17Δ113::MYC-HA or HD-Zip II::CFP was transformed alone. However, a clear signal was registered when ATHB17Δ113::MYC-HA was co-transformed with the constructs expressing HD-Zip II::CFP. No interaction was detected between HD-Zip I protein and ATHB17Δ113 (Figure 6B). These results confirm that when expressed in maize, ATHB17Δ113 can form heterodimers with maize HD-Zip II proteins and, therefore, has the potential to affect activities and pathways associated with maize HD-Zip II proteins.

### ATHB17Δ113 Lacks the Ability to Function as a Transcriptional Repressor

**Figure 7:**
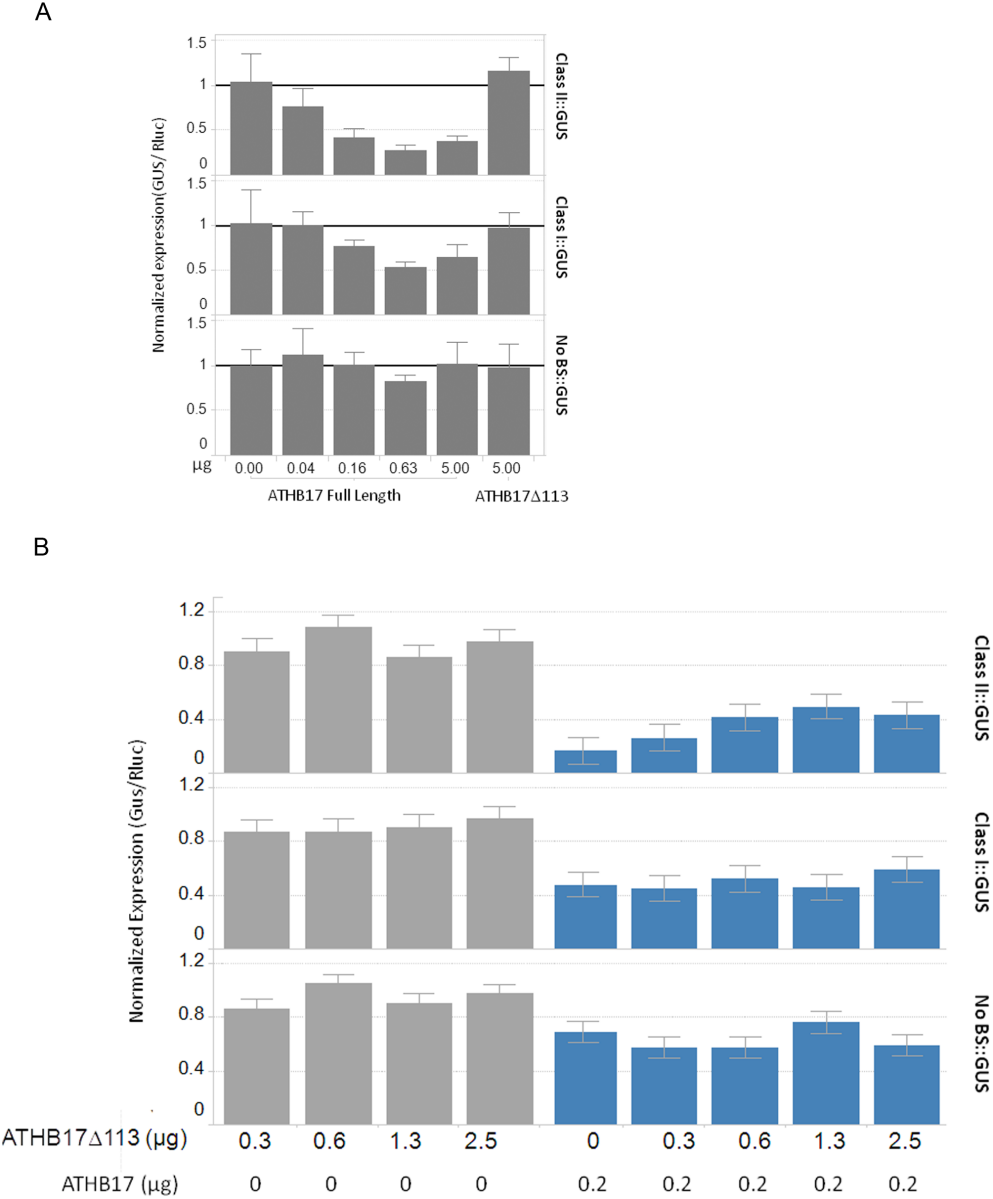
Full- length ATHB17 protein functions as transcriptional repressor and ATHB17Δ113 can relieve repression caused by full-length ATHB17 protein. Maize mesophyll protoplasts were transformed (A) with 4 µg cells of reporter (Class II::GUS, Class I::GUS or No BS::GUS) and 0–5 µg cells of effector (Full-length ATHB17) or 5 µg of ATHB17Δ113 and *Renilla* luciferase (B) with 4 µg reporter (Class II::GUS, Class I::GUS or No BS::GUS), 0–5 µg ATHB17Δ113, and 0 (grey bars) or 0.2 µg (blue bars) of ATHB17 full length. DNA amounts are per 320,000 cells. After 18 h, cells were assayed for GUS and luciferase expression. GUS values were divided by luciferase internal control values for each well and normalized to respective GFP samples. Bars are means and error bars represent 1 SD.

Since ATHB17Δ113 lacks a large portion of the repression domain including the putative EAR-like motif, it should not be able to act as a transcriptional repressor. To determine whether ATHB17Δ113 protein has functional activity as a transcriptional repressor or activator in maize, we established a system that allows for detection of both repression and activation of reporter gene expression in maize mesophyll protoplasts. The transcriptional activity of ATHB17Δ113 was evaluated using two reporter constructs consisting of the GUS gene and either a Class I or a Class II binding sequence positioned between the 35S (−45) minimal promoter and the e35S double enhancer. A control reporter construct contained neither a Class I nor a Class II binding sequence. Transcription was measured by co-transforming increasing amounts of ATHB17 expression plasmid with a constant amount of the reporter plasmid (Figure 7A). Repression of the class II promoter was observed with as little as 40 ng of ATHB17 expression plasmid and was dose-responsive up to 630 ng of the ATHB17 expression plasmid. Repression of the class I promoter was observed with as little as 160 ng of ATHB17 expression plasmid and increased at 630 ng. No repression of the control promoter lacking a binding site was observed. Thus, we confirmed that, in maize protoplasts, ATHB17 can repress transcription from promoters containing class II and, to a lesser degree, class I binding sites. Because no additional repression was observed above 630 ng of ATHB17 expression plasmid, we used smaller amounts in subsequent experiments to improve the assay sensitivity.

In contrast to the repression of the GUS expression demonstrated by the full-length ATHB17 protein, no repression or activation of the ClassI::GUS or ClassII::GUS expression was observed when the ATHB17Δ113 plasmid was co-transformed with the reporter plasmids (Figure 7A). This result shows that the repression activity was lost as a result of splicing in maize. Based on these results, we conclude that ATHB17Δ113 lacks the ability to act as a transcriptional repressor.

### ATHB17Δ113 Acts as a Dominant Negative Regulator of Full-length ATHB17 Protein

Although ATHB17Δ113 does not function as a repressor, the protein retains dimerization and DNA binding properties. Therefore, its likely action is to attenuate the activity of endogenous HD-Zip II proteins through a dominant-negative mechanism (Figure S2). The dominant-negative mechanism can occur through formation of non-functional homodimers or heterodimers with reduced DNA-binding activity or through competition for DNA-binding [46], [47], [48]. These mechanisms naturally occur and have been described in plants. For example, JAZ protein (JASMONATE ZIM-domain protein, a key repressor of Jasmonic acid signaling) has no DNA-binding domain, but can form heterodimers with the MYC2 transcription factor, a transcriptional activator. As a result, the complex between the JAZ and MYC2 proteins is transcriptionally inactive, and can repress the genes that are regulated by MYC2 [49]. Formation of such competitive non-functional heterodimers with reduced DNA-binding affinity also has been described for Arabidopsis little zipper proteins, which can form heterodimers with HD-Zip III proteins. The heterodimers lack the ability to bind DNA and can reduce HD-Zip III protein activity [50]. Although formation of dimers with reduced DNA-binding activity is the most described mechanism thus far, accumulating evidence suggests that heterodimers containing DNA-binding domains but lacking activation domain(s) might reduce transcriptional activity as well as compete for DNA-binding with functional dimers [48].

To evaluate the ability of ATHB17Δ113 to act as a dominant negative regulator, we first examined the ability of ATHB17Δ113 to relieve repression activity of the full-length ATHB17 protein. We used the maize protoplast system described above to co-transform the reporter construct with 0.20 µg of the full-length ATHB17 plasmid and increasing amounts of ATHB17Δ113 plasmid (Figure 7B). Repression of the reporter gene expression caused by the full-length ATHB17 was gradually relieved as increasing amounts of ATHB17Δ113 were added. This observation suggests that ATHB17Δ113 has the ability to act as a de-repressor with a dominant-negative effect on expression of the reporter gene. This dominant-negative effect was only observed with the reporter containing a class II binding sequence, while no significant change in the level of expression was detected with the reporter containing class I binding sequence, or the reporter containing no class I or class II binding sequences. Since we observed lower affinity of binding to the class I sequence in the *in vitro* assay and an overall smaller effect on expression when the reporter construct contained a class I sequence, we hypothesize that ATHB17 may be most active against genes with class II binding sequences in the promoter regions.

### ATHB17Δ113 Acts as a Dominant Negative Regulator of Maize HD-Zip II Proteins

**Figure 8:**
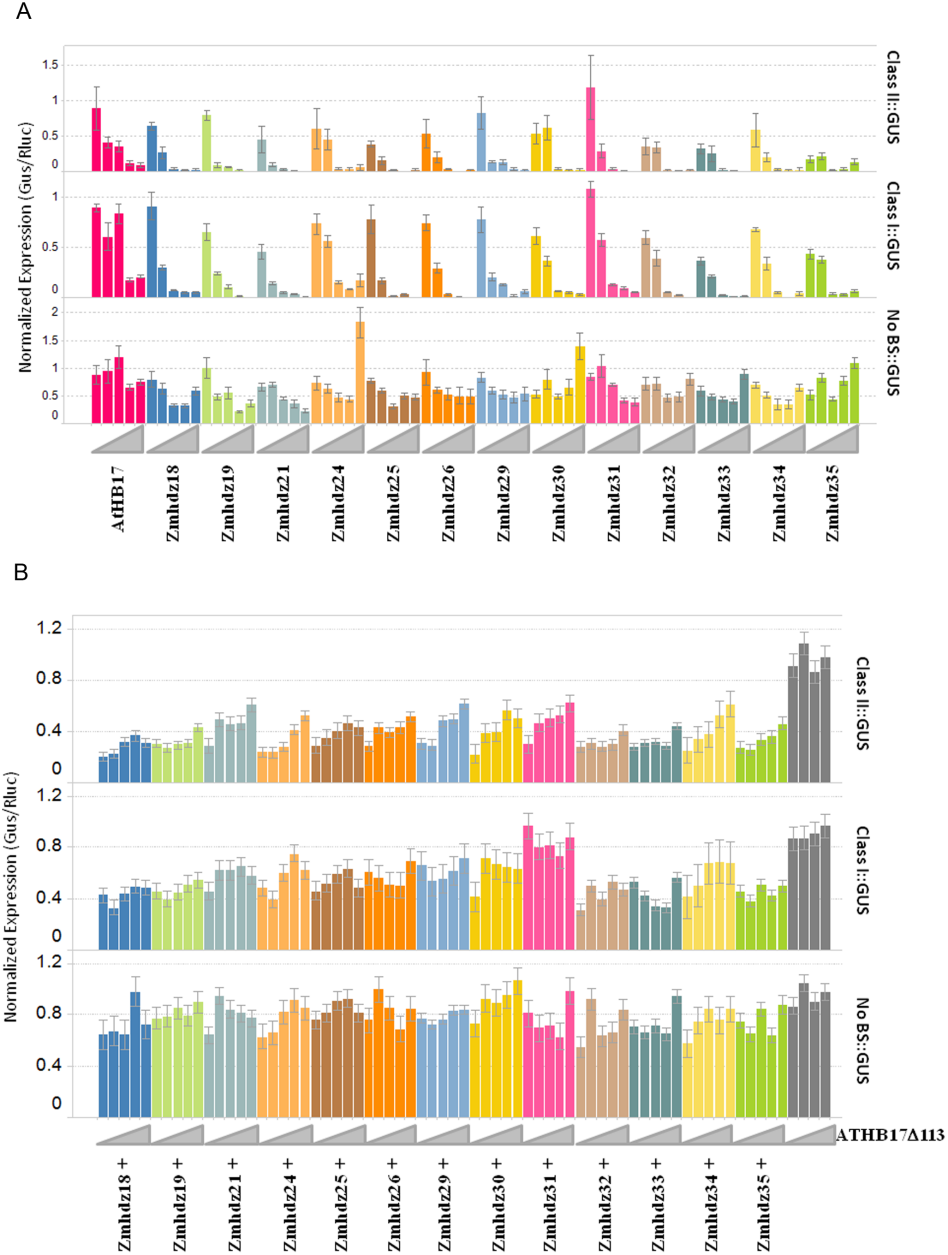
Maize HD-Zip II proteins function as transcriptional repressors and ATHB17Δ113 relieves the repression caused by endogenous maize HD-Zip II proteins. (A) Cells were co-transformed with 4 µg/320,000 cells of each reporter (right side label) and an increasing amount of each HD-Zip II family member (left-to-right for each color: 0.008; 0.04; 0.2; 1; 5 µg/320,000 cells). Error bars are 1 SD. (B) Cells were triple-transformed with 4 µg/320,000 cells of each reporter (right side label), 20 ng of each HD-Zip family member and an increasing amount of ATHB17Δ113 (left-to-right for each color: 0.0, 0.3; 0.6; 1.3; 2.5 µg/320,000 cells). For a given reporter, HD-Zip II, and concentration of ATHB17Δ113, the calculation of standard error relies on the number of biological replicates and the estimated error variance derived from a one-way ANOVA.

Because ATHB17Δ113 can form heterodimers with maize HD-Zip II transcriptional factors and bind to the same DNA sequence, ATHB17Δ113 could be expected to act as a dominant negative regulator of endogenous maize HD-Zip II transcription factors. To evaluate this possibility, we cloned 13 members of the maize HD-Zip II family and tested their activity in leaf protoplasts as described above and in “Material and Methods”. All 13 HD-Zip II proteins evaluated in our study repressed GUS expression with both class I and class II binding sequences in a dosage dependent manner (Figure 8A). This observation suggests that maize HD-Zip IIs act as a transcriptional repressor *in vivo*. To determine whether ATHB17Δ113 has the ability to act as a dominant negative regulator with maize HD-Zip II proteins, the reporter constructs were co-transformed with 20 ng of each HD-Zip II construct and an increasing amount of the ATHB17Δ113 expression construct. Dose-dependent relief of the HD-ZipII repression activity by increasing amounts of ATHB17Δ113 DNA was observed for all tested HD-Zip II proteins when the reporter contained a class II binding sequence in the promoter region (Figure 8B). In contrast, when the reporter construct contained a class I binding sequence or no binding sequences (control construct), no consistent dosage-dependent effect of ATHB17Δ113 on the reporter expression was observed. We conclude that the ATHB17Δ113 protein can act as a dominant-negative regulator of endogenous maize HD-Zip II proteins that repress transcription of genes with promoters containing class II binding sites. This result further indicates that the mechanism of dominant-negative regulation exerted by ATHB17Δ113 should include a competition for class II DNA-binding sites.

### Expression of ATHB17Δ113 in Maize Leads to Increased Ear Weight at Early Reproductive Stages

We investigated whether the expression of ATHB17Δ113 leads to any changes in maize plant development as HD-Zip II proteins are implicated in plant growth and reproductive development [26], [51]. Field studies were conducted over two years in Illinois, USA. Standard agronomic practices (SAP) characteristic of corn production in the region were used to prepare and maintain plots. To determine whether ATHB17Δ113 affected plant growth and development in maize, several parameters were measured for two transgenic events in three hybrids. Because these are quantitative traits that may be subject to environmental variations, we analyzed all of our data across years and hybrids to maximize statistical power. As shown in Table 2, there were no significant changes in the duration of time to the reproductive stage between transgenic events and their corresponding control, as evidenced by the number of days to anthesis and silking and calculated number of days between anthesis and silking (ASI). These results suggest that ATHB17Δ113 does not impact timing of maize development. There was, however, a significant increase in the ear weight at silking (the R1 development stage) in both transgenic events, as compared to the respective control plants (Table 3). There was no observed increase in either stover or total dry matter accumulation at R1. The increase in ear weight without a concurrent increase in vegetative biomass also is evident in the increase in partitioning to the ear (Table 3).

**Table 2:** Phenology of *ATHB17* events and control.

Phase of Development	Event	Mean(days)	Control mean(days)	Delta(days)	% Delta	P-value	N
Planting to 50% silking	Event 1	58.4	58.5	−0.1	−0.2	0.299	110
	Event 2	58.4	58.5	−0.2	−0.3	0.067	110
Planting to 50% anthesis	Event 1	58.1	58.3	−0.2	−0.3	0.080	110
	Event 2	58.2	58.3	−0.1	−0.1	0.548	110
Anthesis silking interval	Event 1	0.26	0.25	0.02	6.1	0.865	111
	Event 2	0.09	0.25	−0.16	−63.9	0.075	111

Two independent *ATHB17* events in three hybrids were used in physiological studies conducted in 2011 and 2012 under standard agricultural practices (SAP) for corn production in the Central Corn Belt. The number of days to 50% silking and anthesis were measured and the number of days between anthesis and silking was calculated (ASI) each year for physiological studies conducted under standard agronomic practices conditions. Differences in phenology between *ATHB17* events and control were determined using an across year combined analysis using a mixed model ANOVA. N denotes the number of data points included per entry in the statistical analysis. Number of event data points were within ±3 of control data points. Results for individual hybrids per year are shown in Table S2.

**Table 3:** Dry matter accumulation in *ATHB17* events and in control at R1 development stage.

Trait Name	Event	Mean	Control mean	Delta	% Delta	P-value	N
Ear dry weight (g/m^2^)	Event 1	99.2	93.1	6.2	6.6	0.006	109
	Event 2	99.7	93.1	6.6	7.1	0.003	109
Stover dry weight (g/m^2^)	Event 1	993.2	956.6	36.6	3.8	0.114	108
	Event 2	951.4	956.6	−5.1	−0.5	0.804	108
Total dry weight (g/m^2^)	Event 1	1069	1052	16.8	1.6	0.482	110
	Event 2	1055	1052	3.1	0.3	0.892	110
Ear partitioning coefficient	Event 1	0.089	0.086	0.003	3.9	0.085	110
	Event 2	0.091	0.086	0.003	5.6	0.013	110

Two independent *ATHB17* events in three hybrids were used in physiological studies conducted in 2011 and 2012 under standard agricultural practices (SAP) for corn production in the Central Corn Belt. Data shown is an across year combined analysis using a mixed model ANOVA of dry matter accumulation data collected at R1. Ear partitioning coefficient was calculated by dividing ear dry weight by total dry weight and analyzed as described above. LSmean for the events and wild type control plants are shown in the table with respective delta, % delta and P-value. N denotes the number of data points included per entry in the statistical analysis. Number of event data points were within ±3 of control data points. Results for individual hybrids per year are shown in Table S3.

In maize, the sink size is determined by environmental and genetic factors around the R1 stage of development [52], [53]. A tight genetic link has been suggested between sink size at flowering and final grain yield [54]. An increase in sink size and partitioning of assimilates to the ear can lead to increased yield if plant growth and development is supported by sufficient source during grain filling [55], [56]. Environmental conditions during grain filling are critical for realization of yield potential [57], [58], [59], [60], and contribute to the variability in measurements of yield. Hence, increased ear weight at R1 provides an opportunity for increased yield, but the realization of this opportunity depends upon the environmental conditions during the grain filling period.

### Maize HD-Zip II Genes are Expressed in Ear Inflorescence and may be Involved in Growth Regulation

Based on the molecular mechanism for ATHB17Δ113 proposed above, it is likely that expression of ATHB17Δ113 impacts maize ear growth by interfering with the functions of endogenous maize HD-Zip II proteins. Several HD-Zip II proteins have been implicated in regulation of growth and development of the meristematic tissue in Arabidopsis [61] including the recently identified JAIBA protein that was shown to regulate growth and development of the inflorescence meristem [26]. To investigate whether the HD-Zip II proteins might be involved in regulation of ear growth in maize, we evaluated expression patterns of maize HD-Zip II genes in two different maize hybrids across several developmental stages in field-grown plants. The sampled tissues included leaf, leaf sheath, stem, ear inflorescence, tassels, ear, ear leaf sheath, cob, and kernels.

**Figure 9:**
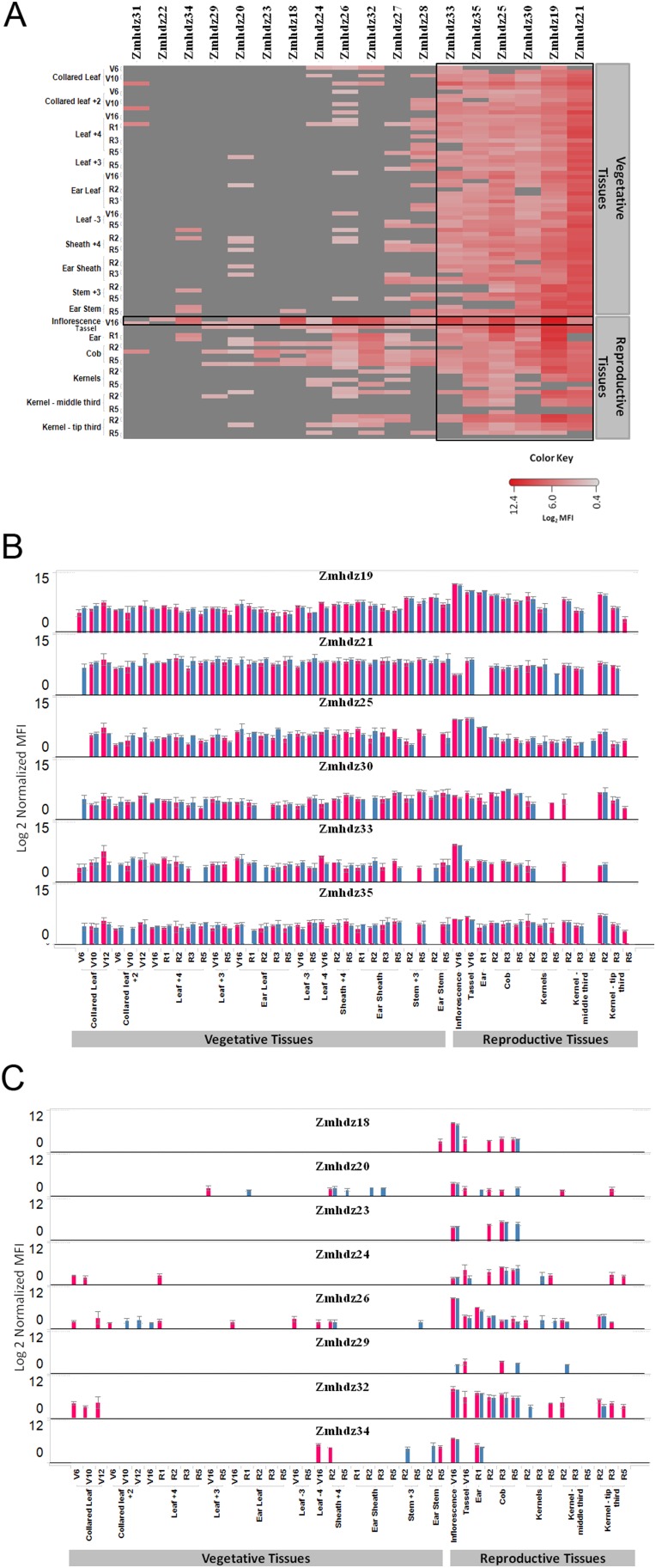
Expression pattern of endogenous HD-Zip II transcripts in maize. (A) Heat map representation of endogenous HD-Zip II transcript expression across tissues and developmental stages in two maize hybrids. The red color gradient shows expression values represented on a Log2 scale normalized median fluorescence intensity (MFI); a darker shade indicates a higher expression level. Grey color represents that expression was below background. For each of the listed tissue and development stages two hybrids (NH6214 and NN6306) are shown in the order that hybrid NN6306 is the first one. (B) Constitutive expression profiles of maize HD-Zip II genes. (C) Reproductive tissue specific profiles of maize HD-Zip II genes. Pink bar represents NN6306 and blue bar represents NH6214. Each panel shows the expression profile for the indicated HD-Zip II gene. The error bars are +/−1 stderr.

The level of transcript in each tissue sampled was determined using the Quantigene 2.0 assay. The assay allowed for the simultaneous measurement of multiple RNA targets, and enabled analyses of the relative expression levels of each HD-Zip II gene. As shown in Figure 6A, maize HD-Zip II genes were found to be expressed in all tissue types and developmental stages similar to what was observed in rice [20] and Arabidopsis [13], [22], [25], [26]. The number of HD-Zip II genes expressed in each tissue and the observed level of expression varied among HD-Zip II genes as shown in Figure 9A. Six genes (Zmhdz19, 21, 25, 30, 33 and 35) were generally expressed in all tested tissues and developmental stages (Figure 9B), suggesting that they are expressed constitutively and, possibly, that they serve a more general role in the regulation of plant growth and development. Eight HD-Zip II genes (Zmhdz18, 20, 23, 24, 26 29, 32 and 34) were predominantly expressed in reproductive tissues (Figure 9C), suggesting that they might be actively involved in regulation of plant reproductive development. The maize ortholog of ATHB17, Zmhdz18, was predominantly expressed in ear inflorescence and cob tissue, but not in kernels or the whole ear. This expression pattern is very similar to the expression of the rice ortholog, OsHox3, suggesting that the genes may have similar functions [20]. Based on their expression patterns, it is hypothesized that many maize HD-ZipII genes may play a role in developmental regulation during a specific period in plant development, as suggested for ATHB17 [31].

Transcripts for a greater number of the HD-Zip II genes were detected in the ear inflorescence than in other maize tissues. Previously, we have shown in a protoplast system that all maize HD-Zip II proteins are repressors of transcription and that ATHB17Δ113 can modulate HD-Zip II functions and relieve the repression when co-expressed in the same tissue. Since ATHB17Δ113 is constitutively expressed in maize and its expression leads to an increase in ear size at silking, it is hypothesized that this phenotype could be a result of ATHB17Δ113 modulating the activity of maize HD-Zip II genes that are expressed in the ear inflorescence. Changes in the activity of maize HD-Zip IIs could lead to changes in the expression levels of their targets. Although no direct targets other than HD-Zip IIs were reported for over-expression of full-length ATHB17 in Arabidopsis plants [31], ATHB17 may have small effects on the expression of its targets. Because ATHB17Δ113 only partially inhibits the repression activity of endogenous maize HD-Zip II proteins, its effect on global transcription in maize would be expected to be very small.

### Expression of ATHB17Δ113 did not Lead to Major Changes in the Ear Inflorescence and Ear Transcriptome

Because the ATHB17Δ113 expressing events have increased R1 ear biomass and because endogenous HD-zip II genes are all expressed in developing ears, we hypothesized that ATHB17Δ113 may influence the ear phenotype at R1 by modulating the activity of HD-zip II transcription factors. To evaluate this, we examined global transcriptome sequences in both ear inflorescence and early ear. We performed transcriptome sequencing using the Illumina HiSeq™ 2000 platform (Illumina, San Diego, CA) on two events in one germplasm.

Each sample had between 12 million and 25 million reads with an average of about 18 million reads. Reads were mapped to the B73 public transcriptome (version 5a). On average, 29% of the reads mapped uniquely to a single transcript, 56% mapped to more than 1 but fewer than 11 transcripts, 1% mapped to more than ten transcripts, and 14% did not map to any transcript. The reference transcriptome consisted of 136,770 transcripts. From the reference transcriptome, 76,612 transcripts were expressed (at least one count) in at least one sample, and 60,158 transcripts were not expressed in any sample. DESeq normalization scale factors ranged from 0.59 to 1.41. No samples were omitted due to quality concerns. As expected, many genes were differentially expressed between the V16 [62] ear inflorescence and R1 ear in the control plants (8961 transcripts; p<0.0001) and similar changes were observed for ATHB17Δ113 expressing transgenic events. However, we observed very few transcripts that were differentially expressed between the ATHB17Δ113 expressing transgenic events and the control in either of the tissues. We identified 11 transcripts that were differentially expressed (raw_p≤0.0001) in event 1, and 24 transcripts that were differentially expressed in event 2 in ear inflorescence (Table 4). In ear tissue, 173 transcripts were differentially expressed in event 1 and five in event 2. To identify transcriptional changes that are most likely to be relevant to the phenotype, we looked for changes in transcript levels that were common to both events. Only seven transcripts showed differential expression (raw_ p≤0.0001) in both events in the ear inflorescence (Table 5) and two transcripts showed differential expression in ear (Table 6). Functional assessment of transcript sequences by BLAST analysis indicates the differentially expressed genes in ear inflorescence have homology to plant Hsp22 heat shock protein, a protein containing RNA Recognition Motif (RRM), a BAG protein, and few hypothetical proteins. Small heat shock proteins can function as chaperones *in vitro* [63]. In maize, mitochondrial hsp22 has been suggested to protect mitochondria against heat stress [64]. RRM-containing proteins are involved in regulation of post-transcriptional gene expression [65]. BAG proteins are multifunctional and are hypothesized to be involved in cell regulation during growth, development, or stress response. Expression of BAG protein homologs was observed in a variety of tissues including developing flowers and ovaries in several plant species [66]. However, the biochemical pathways they may impact and specific functions of different family members are not yet well characterized in plants. Two transcripts were up-regulated in ear tissue of transgenic events (Table 6). One of the transcripts is annotated as glycerophosphodiester phosphodiesterase activity protein (GPPD). GPPD proteins are shown to play an important role in cell wall organization [67]. Cell wall organization is critical during cell expansion during growth. The second is annotated as a putative calmodulin-binding family protein which can be involved in transcriptional regulation [68]. It is not yet clear what role these proteins may play in ear growth and development in maize.

**Table 4:** The number of differentially expressed genes in ear inflorescence and ear of *ATHB17* events relative to control.

Tissue	No. of differentially expressed genes			
		Event 1	Event 2	Overlap
Ear Inflorescence	Up	4	15	1
	Down	7	9	6
Ear	Up	159	3	2
	Down	14	2	0

Table shows number of genes differentially expressed in individual event (raw p≤0.0001) and common between events (overlap).

**Table 5:** List of differentially expressed genes in ear inflorescence in common between two *ATHB17* events.

Gene_ID	Event 1			Event 2			
	FC	RawP	FDRP	FC	RawP	FDRP	Annotation
GRMZM2G007729	−2.77	2E-06	1E-01	−2.66	2E-06	5E-02	Heat shock 22 kDa protein
GRMZM2G008611	−1.56	4E-06	1E-01	−1.44	2E-05	2E-01	Hypothetical protein containing RRM motif
GRMZM2G051135	−2.40	4E-05	4E-01	−2.71	2E-05	2E-01	Hypothetical protein
GRMZM2G097135	−5.41	6E-05	5E-01	−5.09	7E-05	3E-01	BAG domain containing protein
GRMZM5G823696	−5.29	5E-05	4E-01	−6.32	4E-05	2E-01	Hypothetical protein
GRMZM5G841343	−2.23	2E-05	3E-01	−2.18	2E-05	2E-01	Hypothetical protein
GRMZM5G845499	1.91	4E-05	4E-01	1.91	4E-05	2E-01	Hypothetical protein

Table lists the fold change (Fc), raw and fdr p-values for common genes in each event.

**Table 6:** List of differentially expressed genes in ear in common between two *ATHB17* events.

Gene_ID	Event 1			Event 2			
	FC	RawP	FDRP	FC	RawP	FDRP	Annotation
GRMZM2G059129	2.23	1.28E-07	0.0034	1.65	2.66E-05	0.925423	Glycerophosphodiester phosphodiesterase activity
GRMZM2G168222	2.16	5.78E-07	0.011072	1.70	4.12E-05	0.925423	Putative calmodulin-binding family protein

Table lists the fold change (Fc), raw and fdr p-values for common genes in each event.

Although HD-Zip II proteins have been shown to regulate the expression of each other [6], [8], we did not observe any significant changes in transcript levels for any of the endogenous maize HD-Zip II genes in either tissue (raw_p<0.0001). Overall, the observed effects of ATHB17Δ113 on the maize ear inflorescence and ear transcriptome were very small. This is not unexpected, since the ATHB17Δ113 does not have direct repression activity and can only act through modulation of the activity of other HD-Zip IIs and the overall effect could be very subtle.

## Conclusion

ATHB17 protein functions as a transcriptional repressor and contains an EAR-like motif within the repression domain. Similar to other HD-Zip II proteins, ATHB17 requires a functional homeodomain and leucine zipper for transcriptional repression. The cellular localization of ATHB17 could be regulated through the N-terminus which is not present in other members of the family. Introduction of the *ATHB17* coding sequence into maize leads to the expression of ATHB17Δ113, a truncated protein lacking a repression domain and transcriptional repression activity. ATHB17Δ113 forms homodimers which bind to the consensus DNA binding sequence, and heterodimerizes with endogenous maize HD-Zip II proteins. We show that the protein can disrupt HD-Zip II mediated transcriptional activity and is active in protoplasts as a dominant-negative regulator of the transcriptional repression activity of endogenous maize HD-Zip II proteins. Because ATHB17Δ113 lacks a functional repression domain, it is unlikely to have transcriptional activity by itself, and we hypothesize that ATHB17Δ113 may function by altering the activity of the endogenous HD-Zip II proteins. Modulation of the HD-Zip II regulated pathways may in turn lead to changes in the ear inflorescence growth and, ultimately, a larger ear at the early reproductive stage. The transcriptome analysis revealed only a few changes in the expression of native genes in the ear inflorescence and early ear, and no marked changes in HD-Zip II expression were observed. We therefore propose that subtle changes in transcription can affect agronomically important quantitative traits without causing undesirable phenotypic off-types that have been observed in past efforts to improve crop performance by overexpressing transcription factors [69].

## Materials and Methods

### Ethics Statement

Approval to conduct the field study in Unites States was obtained from United States Department of Agriculture. Field studies were performed on private land in Jerseyville (ILW1 and ILW2) and Wrights (ILWR), Illinois, United States of America (GPS co-ordinates for 2011-ILW1 (39.0650, −90.3133), ILW2 (39.0650, −90.3107), ILWR (39.3456, −90.2790) and for 2012-ILW1 (39.0695, −90.3074), ILW2 (39.0695, −90.3074), ILWR (39.3381, −90.2856) for which Monsanto Company has lease agreement with land owner. Field studies did not involve any endangered or protected species.

### Comparative Sequence and Phylogenetic Analysis

Protein sequences for ATHB17 orthologs and paralogs were identified by searching public databases and were aligned using CLUSTALX [70]. Sequences belonging to the HD-Zip II α subclass (ATHB2, ATHAT1 and ATHAT2) were used as an outgroup. Transmembrane domains in ATHB17 were identified using TransMem+ (Wisconsin Package Version 10.3 software suite (Accelrys Inc., San Diego, CA, USA)) using the default settings.

The protein sequence for ATHB17 was analyzed for potential localization signals using pSort prediction software [36]. The software uses two rules to determine potential nuclear localization signals. The first is the identification of 4 residue pattern composed of basic amino acids (K or R), or composed of three basic amino acids (K or R) and H or P. The second is the identification of a bipartite signal composed of 2 basic residues, 10 residue spacer, and another basic region consisting of at least 3 basic residues out of 5 residues. The program identified a 7 residue pattern starting at amino acid 136. No bipartite signals were identified. A second potential nuclear targeting signal was identified by manually searching for highly basic regions. The region of amino acids 187–194 was identified composed of 5 basic residues out of 8.

### Transcriptional Repression Assay in Arabidopsis Protoplasts

The *35S:: chloramphenicol acetyltransferase* (*CAT*) gene was described previously [38]. The cDNA sequence encoding full-length ATHB17, ATHB17 variants, or ATHB2 was cloned downstream of *CaMV 35S* promoter and a translational enhancer from the 5′-leader of Tobacco mosaic virus [71]. The promoter sequence for *HAT1* (1821 bp) was amplified by PCR and was cloned upstream to the *GUS* (*beta-glucuronidase*) reporter gene. The *pHAT1*::*GUS* reporter gene was used in all transcription repression assays because the basal level of expression and magnitude of repression was better than that observed with *pHAT2*::*GUS*. The transcriptional terminator from the *A. tumefaciens NOS* gene was used in all reporter and effector plasmids [71]. The *ATHB17* (AT2G01430) and *ATHB4* (AT4G16780) clones were PCR amplified from a diversified cDNA library created using mRNA from several different Arabidopsis tissues that was generated at Mendel Biotechnology, Inc. The *ATHB17* PCR product amplified at Mendel contained an M227I substitution compared to the peptide sequence deposited at The Arabidopsis Information Resource (TAIR). This Mendel-derived sequence was used to construct all the variants described in this study. *ATHB17* derivatives presented here were generated by standard techniques of molecular cloning [72] and the In-Fusion PCR cloning system (Clontech, Mountain View, CA).

Protoplasts were prepared from whole leaves of three- to four-week old Arabidopsis plants using an enzyme solution containing cellulase (Research Products International Corp, Mount Prospect, IL) and macerozyme (SERVA Electrophoresis GmbH, Islandia, NY) to remove cell walls [38]. For the transcriptional repression assay, 200 µl of protoplast cells were co-transformed with 10 µg of the *Gal4UAS*(4X)::*GUS* reporter gene construct with either 10 µg of *CAT*, *ATHB17*, or *ATHB17* variant constructs. Plasmid DNA used for co-transformation was prepared using EndoFree Plasmid Maxi kits (QIAGEN, Valencia, CA). DNA was introduced into protoplasts by incubating protoplasts with 40% PEG 4000 for 30 min. After removal of PEG, protoplasts were kept in the dark at room temperature for 18 to 20 h. Protoplasts were then lysed in 1x cell culture lysis buffer (Promega, Madison, WI) and incubated with 1 mM 4-methylumbelliferyl-beta-D-glucuronide (Gold Biotechnology Inc., St. Louis, MO) solution at 37°C for 1 h. GUS activity was quantified by taking fluorescence measurements using a Synergy HT Microplate Reader (BioTek, Winooski, VT). Three biological replicates were performed and GUS measurements for each construct were averaged.

### Generation of Maize Transgenic Events and Expression Analysis

A binary plasmid vector for transformation into maize was prepared containing the rice actin promoter, a leader and an intron driving the expression of full length *ATHB17* coding sequence and 3′polyadenylation sequence. Primer sequences used to amplify *ATHB17* coding sequence are as follows: ATHB17 F1-ATATATATggtaccATGATAAAACTACTATTTACGTAC and ATHB17 R1- ATATATATgggcccCGACTCAACGATCACGCTCTTG. Transgenic maize events expressing ATHB17 were developed through *Agrobacterium*–mediated transformation of immature maize embryos based on the method described by Sidorov and Duncan [73]. In brief, immature embryos were excised from a post-pollinated maize ear. After co-culturing the excised immature embryos with *Agrobacterium* carrying the *ATHB17* plasmid vector, the immature embryos were placed on selection medium containing glyphosate and carbenicillin disodium salt in order to inhibit the growth of untransformed plant cells and excess *Agrobacterium.* To confer tolerance to glyphosate the *ATHB17* plasmid vector also contained the selectable marker CP4 *EPSPS* coding region regulated by rice actin promoter, leader, and intron, and a 3′ polyadenylation sequence. Events containing the *ATHB17* cassette were characterized by detailed molecular analyses to select events that contain only one copy of the *ATHB17* cassette.

To determine the expression of *ATHB17* in transgenic events, proteins were extracted from the lyophilized leaf tissues from growth-chamber-grown plants using 1∶50 extraction buffer containing 8 M urea. The samples were homogenized at 1200 rpm for 120 sec at room temperature (RT) using a megagrinder. They were then sonicated with a Model 2510 R-MTH Branson Ultrasonicator (Branson Ultrasonics, Danbury, CT) for 1 min at RT, centrifuged at 2800 rpm at 20°C for 20 min, and the supernatant was collected. Ten µl of sample were loaded into each lane of a 10% Bis-Tris LDS Midi gel (Invitrogen, Carlsbad, CA) at 10 µl/lane as per *NuPAGE Technical Guide*. The proteins were transferred to a nitrocellulose membrane at 100 V constant voltage for 1 hour with 1X transfer buffer containing 20% MeOH and 0.1% antioxidant. The membrane was blocked with 30 ml of WesternBreeze (Invitrogen) block for 2 hours at RT. The membrane was then washed twice with 40 ml ultrapure water, 5 min each time, and probed with primary antibody diluted 1∶1000 in WesternBreeze block for 1 hour at RT with shaking. The membrane was washed thoroughly three times with 30 ml of antibody wash from the WesternBreeze kit for 5 min and probed with 30 ml of secondary antibody solution from the WesternBreeze kit for 1 hour at RT while shaking. The nitrocellulose membrane was then washed three times with 40 ml of antibody wash from the WesternBreeze kit for 5 min each and rinsed twice with 40 ml of ultrapure water for 5 min each. Detection was performed with the WesternBreeze Chemiluminescent Western Blot Immunodetection Kit (Product#WB7106) as per the manufacturer’s instruction.

### ATHB17 Transcript Sequencing

Total RNA was extracted using a Trizol method. Briefly, Trizol reagent (Ambion by Life Technologies, Carlsbad, CA) was added to powdered leaf tissue and mixed by vortexing. Following an incubation at RT, chloroform (Fisher Scientific, Pittsburgh, PA) was added and mixed. Following another incubation at RT, the tube was centrifuged at 12,000×g at ∼4°C for 10 min. The aqueous phase was transferred to a new tube, and the RNA was precipitated with a half-volume of isopropyl alcohol (Sigma, Saint Louis, MO). To pellet the RNA, the tube was centrifuged at 12,000×g at ∼4°C for 20 min. The RNA pellet was washed using 70% (v/v) ethanol and resuspended in DEPC-treated water (Ambion). Poly(A) RNA was enriched using the Ambion MicroPoly (A) Purist MAG Kit (Life Technologies, Carlsbad, CA) as per manufacturer’s specifications. All poly(A) enriched RNA was resuspended in water. Total RNA was quantified using a Nanodrop™ Spectrophotometer (Thermo Scientific, Wilmington, DE) following the manufacturer’s instructions. cDNA was synthesized using SuperScript III Reverse Transcriptase kit (Life Technologies, Carlsbad, CA) according to the manufacturer’s specifications.

A single PCR product (Product A) was generated that was analyzed to determine the nucleotide sequence of *ATHB17* RNA. The PCR amplification of Product A was performed on event 2 *ATHB17* cDNA in a 50 ml reaction volume. Each reaction contained a final concentration of 0.2 μM of each primer and 1X Phusion HF MasterMix Buffer (NEB, Ipswich, MA). The following cycling conditions were followed: 35 cycles at 98°C for 30 sec; 60°C for 30 sec; 72°C for 1 min with an initial denaturation step at 98°C for 30 sec and a final elongation cycle for 10 min at 72°C. Aliquots of each PCR product were separated on a 1% (w/v) agarose gel and visualized by ethidium bromide staining to verify that the products were the expected size. Prior to sequencing, each verified PCR product was purified using an ExoSAP-IT PCR Product Cleanup Kit (Affymetrix, Santa Clara, CA) according to the manufacturer’s instructions.

Sequencing was performed using BigDye terminator chemistry (Applied Biosystems, Foster City, CA). A consensus sequence was generated by compiling sequences from multiple sequencing reactions performed on the PCR product.

### Localization of ATHB17 Full-length and Variants in Arabidopsis and Maize Protoplasts

Arabidopsis mesophyll protoplasts were isolated [74] and transformed using polyethylene glycol (PEG4000). Approximately 1.5×10^5^ protoplasts were transformed with 10 mg of plasmid DNA per test construct for single protein expression. Maize leaf protoplasts were isolated from etiolated maize seedlings as described in Sheen and Bogorad [75] and transformed using PEG4000 [76]. Approximately 3×10^5^ protoplasts were transformed with 12 mg of plasmid DNA per test construct for single protein expression. Plasmid DNA was prepped using the Qiagen (Germantown, MD) Plasmid Mega kit. Protoplasts were incubated a total of 18 to 24 hours at 22°C and analyzed for GFP fluorescence using confocal microscopy. Confocal fluorescence microscopy analysis was performed using a Zeiss LSM510 META Laser Scanning Microscope (Carl Zeiss MicroImaging, Inc., Thornwood, NY) equipped with a Krypton-Argon Ion (488 nm) laser, a green (543 nm) Helium-Neon laser, FITC, and Texas red filter sets. Image acquisition and analysis was performed using LSM 5 Image Examiner, version 4.2 (Carl Zeiss MicroImaging, Inc.), using a 40x water 1.2 numerical aperture objective. Excitation wavelengths used were 488 nm and 543 nm, and emission filters were 500 to 530 nm and 630 to 700 nm to detect GFP and chlorophyll, respectively.

### DNA Binding

Oligonucleotides, CAGA**CAATCATTG**CGGC (Class II), CAGA**CAATTATTG**CGGC (Class I), and CAGCTCAGTCTGACGGC (non consensus) were from Integrated DNA Technologies (IDT, Coralville, IA). The pseudo-palindromic sequences flanked by four nucleotides at the 5′ end and 3′end of the oligo are in bold. The Amine coupling Kit (BR-1000-50) containing N-hydroxysuccinimide (NHS), 1-ethyl-3-(3-dimethyl-amino-propyl)-carbodiimide Hydrochloride (EDC), Ethanolamine, pH 8.5, and Carboxylmethyl dextrane CM5 sensor-chip, Nickel-NTA (Ni-NTA) column, and HiLoad Superdex column were all from GE Healthcare Life Sciences (Piscataway, NJ), Streptavidin was from Thermo Fisher Scientific (Rockford, IL). Amine coupling of Streptavidin and capturing of biotinylated DNA were performed with 10 mM HEPES pH 7.4, 150 mM NaCl, 3 mM EDTA, and 0.005% Tween-20 (HBS-EP). Biacore binding assays were in HBS-EP with 100 µg/ml BSA. Scrubber 2.0c was from Biologic Software Pty Limited (Campbell, Australia). Octet QK running buffer, 1X PBS (10 mM Na_2_HPO_4_, 1 mM KH_2_PO_4_, 137 mM NaCl, 2.7 mM KCl, pH 7.4) was from Roche Diagnostics (Indianapolis, IN). Tween-20 was from Sigma-Aldrich Corporation (Saint-Louis, MO). Streptavidin sensor tips were from Forte Bio, Inc. (Menlo Park, CA) and microplates, black 96 well, were from E&K Scientific Products, Inc. (Santa Clara, CA).

Tag free ATHB17Δ113 was expressed in *E coli* and purified by conventional chromatography. Surface plasmon resonance (SPR) experiments were performed using a Biacore 2000 instrument at 25°C with filtered and degassed HBS-EP buffer. To generate a streptavidin sensor chip by amine coupling, CM5 sensor chip was conditioned with successive 20 sec injections of 0.1 N HCl, 0.1 N NaOH, and 0.5% SDS at 100 µl.min^−1^. The conditioned chip matrix was washed with HBS-EP buffer at a flow rate of 5 µl min^−1^, followed by activation of all four flow cells (Fc) with 35 µl of 1∶1 NHS/EDC (75 mg ml^−1^/11.5 mg ml^−1^). Coupling was achieved with 35 µl injection of 200 µg/ml streptavidin in 10 mM sodium acetate (pH 6.5). Non-reacted carboxylmethyl groups were blocked with 35 µl of 1 M ethanolamine (pH 8.5). Biotinylated DNA (3 nM) in HBS-EP was captured at a flow rate of 5µl min^−1^. The flow cell 1 (Fc1) was without captured DNA target and served as reference surface.

Real time kinetics were performed at a flow rate of 50 µl min^−1^ in HBS-EP with 100 µg/ml BSA as running buffer. Serial dilutions of five concentrations of ATHB17Δ113 (3.7 nM, 11.1 nM, 33.3 nM, 100 nM and 300 nM) were injected. Two injections of buffer were performed before and after protein injections and used as blanks. Each 150 µl protein/buffer injection was followed by a 400 sec dissociation period. The surface was regenerated for subsequent runs with a 10 µl injection of 0.01% SDS. All SPR data analyses were performed by global fitting to a 1∶1 Langmuir binding model using Scrubber 2.0c.

All Octet-QK (Bio Layer Interferometry) experiments were performed in PBS (pH 7.4) supplemented with 0.005% Tween-20 (PBS-T) and carried out at 30°C. Assay samples were in 200 ul per microplate well and agitated at 1000 rpm. Streptavidin sensor tips were hydrated in reaction buffer before use. There are five steps in a typical Octet-QK assay. The initial three steps included a baseline step (1) to continue hydrating the sensor tips, followed by an immobilization step (2) to capture target DNA (6 nM) and an additional baseline step (3). Hydration, baselines and DNA capture steps were each for 10 min. In the last two steps which constitute the actual kinetic reaction, the association step (4) was initiated with samples of ATHB17Δ113 for 10 min and the dissociation step (5) was allowed for 15 min in PBS-T. Binding kinetics of target DNA variants were measured with 30 nM of ATHB17Δ113. Octet QK binding experiments were locally fitted to a 1∶1 binding model using Octet 4.0 software package.

### Protein- protein Interaction

Maize leaf protoplasts were isolated from 12- day-old plants as described in Sheen and Bogorad [75] and transformed using PEG [76]. Approximately 3×10^5^ protoplasts were transformed with a total of 12 µg of Qiagen prepped plasmid DNA 6 µg per expression construct or 6 µg pGEM filler DNA. Protoplasts were incubated 18 to 24 hours at 22°C. Protoplasts were pelleted at 150 g for 3 min and all but 300 µl of incubation buffer was decanted. Pelleted cells were resuspended, 300 µl transferred to 96-deep well plate (#P-DW-500-C, VWR International, Radnor, PA) and centrifuged at 150×g for 3 min. The remaining incubation buffer was decanted, leaving 20 µl. The cells were lysed by adding 100 µl Protein lysis buffer (50 mM Tris pH 7.8, 150 mM NaCl, 1%Triton X-100 (#9002-93-1, Sigma), 1x protease inhibitor Roche complete tablets (#11-836-153-001, Roche Diagnostics Corporation, Indianapolis, IN) and mixing three times every 15 min for 1 hour. The protein lysate was centrifuged at 3000×g for 5 min and soluble fractions were retained for use in Luminex based co-immunoprecipitation (co-IP) assay (Luminex Corporation, Austin, Texas).

Capture antibodies for CFP (D153-3, MBL International Corporation, Woburn, MA), MYC (cat#A190-104A, Bethyl Laboratories Inc., Montgomery, TX), and HA (cat#A190-107A, Bethyl Laboratories Inc.) were covalently coupled to carboxylated fluorescent microspheres (Luminex) according to the manufacturer’s protocol. Luminex two step Carbodiimide coupling protocol was modified as follows: 3.5×10^6^ beads were conjugated to 15 µg of antibody and resuspended in coupling buffer containing 100 mM MES pH 6. Biotinylated antibodies for CFP (#ab6658, Abcam), MYC (#A190-104B, Bethyl Laboratories Inc.) and HA (#A190-107B, Bethyl Laboratories Inc.) were used for detection of interacting prey proteins in the miniaturized sandwich immunoassay. NeutrAvidin R-phycoerythrin was used as reporter (A2660, Invitrogen). Protein expression for ATHB17MYC::HA dual tag and CFP constructs were detected using the miniaturized sandwich immunoassay and co-IP method as described by Qi et al. [77]. Fifty µl lysate was aliquoted to either 3 or 4 different wells in a 96-well clear flat-bottom plate (#171-025001, Bio-Rad Hercules, CA). To each sample, 50 µl of conjugated beads (5000 beads per well diluted in PBS-T 0.05% Tween and 0.5% BSA) was added to each appropriate well and incubated for 30 min with shaking. Forward and reverse Co-IPs were performed with conjugated beads mentioned above. Beads were captured for 2 min and washed 3 times with PBS-T. Biotinylated antibodies at (1∶1000) were added to appropriate wells. The plate was incubated on a shaker for 30 min followed by bead capture and wash. A 100 µl of reporter NeutrAvidin R-phycoerythrin at (1∶1000) was added to each well and incubated 30 min on a shaker with subsequent washing. Each well was resuspended in 100µl of PBS-T buffer before plates were analyzed with FLEX MAP 3D system (Luminex Corporation). Approximately 100 beads were measured per sample to determine the median fluorescence intensity (MFI).

Samples of mock transformed protoplasts, bait alone, and prey alone were used to determine the background signal of the Luminex assay. The mean and standard deviation of the MFI of these negative controls were calculated based on biological duplicates or triplicates. The mean+3 sigma was used as cut-off to distinguish background signal from positive signal. Signal above the +3 sigma background was designated as positive signal. Comparisons were performed using ANOVA t-test (JMP 8, SAS Institute, Cary, NC).

### Yeast-two-hybrid Screen

*ATHB17* full-length and multiple fragments were used as bait in a yeast-two-hybrid screen performed using ULTImate Y2H™ by Hybrigenics (Paris, France). cDNA encoding full-length *ATHB17* was cloned into pB27 and pB29 (LexA- C and N terminal fusions), EAR motif region 1–91 into pB29 (LexA N-terminal fusion), Homeodomain region 128–234 into pB27 (LexA C-terminal fusion), Leucine zipper and Homeo-domain region 114–275 into pB27 (LexA C-terminal fusion). These bait constructs were used to screen a random-primed cDNA library (MEGA_RP1) prepared from maize RNAs from callus tissue, etiolated seedlings, V3 seedlings, ear inflorescence, developing kernels, and ear leaf. 111–139 million clones (11–14 fold coverage of the library) were screened. cDNA fragments corresponding to positive 330 “prey” clones were amplified by PCR and sequenced at their 5′ and 3′ junctions. The resulting sequences were searched against Monsanto’s proprietary database and assigned a quality score (A through F) which was indicative of the confidence of interaction.

### Transcriptional Repression Assay in Maize Protoplasts

Maize protoplasts were transformed as described above. 53,000 cells were transformed for each replicate. Each treatment was tested in four technical replicates per transformation. Biological replicates consisted of identical treatments tested in protoplasts isolated on different days. Cells were transformed with up to 6 constructs: varying amounts of 1–2 effector DNA constructs (amounts are indicated in each figure), a constant amount of one GUS reporter construct, varying amounts of GFP, and constant amounts of two internal controls. GFP fluorescence was not used as a reporter in these assays; GFP was included to balance both the total amount of DNA and the amount of 35S-equivalent expression in each cell. Hence, when titrating effects of the various effectors, differences in amount of DNA transformed per well were normalized by co-transforming with GFP so that each well contained no more than 10 µg/320,000 cells of DNA. After addition of DNA, the total volume in each well was normalized by the addition of ddH_2_O. Internal control constructs expressing firefly and *Renilla* luciferase, were used to normalize for normal well-to-well variations in transformation efficiency. Following background subtraction using “cells only” samples, data was normalized by dividing GUS counts per second per well by relative luciferase units per well.

### Physiological Phenotypes at R1

Field experiments were planted in Illinois, USA, in 2011 and 2012. Two different ATHB17Δ113 expressing events were compared to control plants in the context of three different Monsanto elite hybrids (NH6214, EXP257, NN6306). When insect protection and glyphosate tolerance traits were included, both test and control entries contained the same traits. NH6214 and EXP257 were tested in 2011 and NH6214 and NN6306 were tested in 2012. The experiment was a randomized complete block design (RCBD) with 10 replicates and two testers blocked separately within the trial in 2011. The experiment was a group unbalanced block design (GUBD) with 18 replicates and two testers blocked randomly within the trial in 2012. Experimental units consisted of three 2-row plots (21.5 ft long×15 ft wide) blocked together for a total of 6 rows for each entry in the study. Standard agronomic practices (SAP), which are characteristic of corn production practices for the region, were used to prepare and maintain each field site. Maintenance pesticides were applied as needed and all maintenance operations were performed uniformly over the entire production area at a given field site. Plant development was followed on 10 tagged plants per plot. Days to reproductive developmental stages were assessed from planting until 50% of the plants reached anthesis and silking. Anthesis-silking interval was calculated by subtracting the number of days to 50% anthesis from the number of days to 50% silking.

Biomass samples were collected when plants reached the R1 growth stage. In 2011, ten plants from a given area were harvested for biomass and the area measured; while in 2012, all plants from a 1-m row were sampled for biomass and the plant number was counted. Plants were harvested by cutting the stalk at soil level and separating each plant into its component parts, including leaf blades, stalks with leaf sheaths, and ear shoots (with husk and shank). All plant parts were dried at approximately 70°C until a constant weight was achieved. Biomass of each plant component was recorded. Stover biomass was calculated by summing biomass of leaves and stalks. Total biomass was calculated by summing biomass of stover and ear shoots. Data from 2011 was presented as g/m^2^, while data from 2012 was converted via covariate analysis into plant number adjusted g/m^2^. The ratio of ear weight to total weight was calculated at R1 to determine relative early partitioning of assimilates to the developing ear.

Data were analyzed with the MIXED procedure of SAS 9.2 software (SAS Institute, Cary NC). Outliers were identified using the deleted studentized residual (DSR) method, and outlier data points were discarded before further statistical analysis. Each data set consisted of events 1 and 2 and the corresponding control. Entries were analyzed across environments by using a mixed effects model. For model fitting, hybrid entry was considered a fixed effect, while environment, rep within environment, the environment x entry interaction and the residual (experimental error) were considered random effects. Environment was defined as the combination of set within year and field. The statistical model used was:
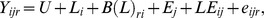
where Y_ijr_ = Observation from the j-th entry within the r-th rep at the i-th environment, U = overall mean effect, E_j_ = Effect of the j-th entry, L_i_ = random effect of the i-th environment, B(L)_ri_ = random effect of the r-th replicate within the i-th environment, LE_ ij_ = random interaction effect between the i-th environment and the j-th entry, and e_ ijr_ = experimental error (residual) corresponding to the observation Y_ijr_.

Point estimates of the mean of event and control, difference between event and control means (delta), delta as a percentage of control mean (percent delta), and p-value for the test of delta were provided for comparing event vs. control across 2011–12 and across hybrids, and by year and by hybrid.

### HD-Zip II Expression Analysis by Quantigene 2.0

Tissue samples were collected from 2012 field study (hybrids NH6214 and NN6306). RNA was extracted using the EZNA RNA Purification Kit (Omega BioTek, Norcross, GA). The transcript expression of 18 HD-Zip IIs was analyzed using Quantigene 2.0. Raw mean fluorescence intensity (MFI) were obtained using the Quantigene 2.0. QC step included assigning 0.5 LOD value for MFI that are below LOD only if more than 50% of the data available. LOD was calculated using the formula LOD = AVG MFI of assay background control wells+3 SD of assay background signals. Each raw MFI was then subtracted with the averaged no template background MFI.

This background subtracted MFI was normalized prior to log transformation. For normalization, the mean of housekeeping gene was computed across all samples (3entries×3replicates). Then a correction/normalization factor was obtained by dividing mean of housekeeping gene by each individual MFI of housekeeping gene (Asparaginase). This correction/normalization factor was multiplied with each individual background subtracted MFI for each “trait” (datapoint within that tissue type) and then Log2 transformed to get the final normalized values for each trait.

### RNA Extraction, RNA-seq Sample Preparation and Sequencing

Tissue samples were collected from the 2012 field study (hybrid NN6306). Total RNA was isolated from three biological replicates for each tissue using TRIzol (Invitrogen) according to the manufacturer’s protocol. RNA quantity was determined with a Nanodrop 8000 spectrophotometer and integrity assessed by Bioanalyzer assay with RIN greater than 6. Two µg of total RNA was used for sequencing library preparation with Illumina TruSeq RNA Sample Prep Kit V2 following manufacturer’s protocol. qPCR (SYBR PCR Master Mix, Applied Biosystems) has been utilized to quantify sequencing libraries. Sequencing was performed with HiSeq2000 sequencing using 50 base pair reads.

### Sequence Alignment, Data Normalization, QC Analysis and Statistical Analysis

The Bowtie application was used for mapping the reads to the public B73 maize transcriptome reference. We allowed up to 2 high quality mismatches for every 28 seed sequence, where “high quality” means an average Phred score greater than 20. Individual reads were mapped to the transcriptome. Reads may map uniquely, to multiple transcripts, or to no transcripts. If a read mapped to more than 10 transcripts, it was ignored. If it mapped to 10 or less, it was assigned equally to all of them. The DESeq algorithm (Simon Anders, EMBL) was then used to normalize the raw counts across all the samples in an experiment. Principal component analysis, hierarchical cluster analysis, and correlation analysis were used for QC measures and identify poor quality samples. Proc GLIMMIX (in SAS) was used to identify differentially expressed genes.

The data discussed in this publication have been deposited in NCBI’s Gene Expression Omnibus [78] and are accessible through GEO Series accession number GSE51742 (http://www.ncbi.nlm.nih.gov/geo/query/acc.cgi?acc=GSE51742).

## Supporting Information

Figure S1 (A) ATHB17 Transcript sequence from transgenic event. (B) Predicted Amino Acid Sequence of the Protein Produced by *ATHB17 RNA* in transgenic event. The generated consensus sequence of ATHB17 transcript from the transgenic event was used in a BLASTX 2.2.23 search of the GenBank_Protein_Preferred database. The predicted amino acid sequence of ATHB17 protein starting with first methionine is shown. Compared to the Arabidopsis ATHB17 protein sequence (GenBank ID 179876107), the ATHB17 predicted protein sequence is truncated in transgenic event; lacking the amino-terminal 113 amino acids and is designated as ATHB17Δ113. (C) *ATHB17* transcription and translation products in transgenic maize events. ATHB17 mRNA sequence identifies truncation of the 5′ region of the ATHB17 coding sequence. This transcript sequence is predicted to produce a truncated protein lacking the first 113 amino acids. (D) Domain structure of ATHB17. ATHB17 coding sequence contains canonical Homeodomain and Leucine Zipper (LZ) domains of HD-Zip family. Homeodomain is required for DNA binding. LZ domain is responsible for homodimerization and hetero-dimerrization with other HD-Zip II proteins. A repression domain is present upstream to the homeodomain. ATHB17 protein expressed in transgenic maize events lacks the first 113 amino acids resulting in deletion of part of repression domain containing EAR-like motif.
(TIF)

Figure S2 **ATHB17Δ113 will function via dominant negative mechanism.** ATHB17 is expressed as truncated protein in maize lacking part of the repression domain. Maize endogenous HD-Zip IIs function as transcriptional repressors. ATHB17Δ113 can interact with endogenous HD-Zip IIs and sequester endogenous proteins from binding to their targets resulting in relief of repression caused by maize HD-Zip IIs. In addition, heterodimer of ATHB17Δ113 and endogenous HD-Zip IIs or ATHB17Δ113 homodimers can compete for DNA binding resulting in altered target expression due to inability to cause active repression.
(TIF)

Table S1 **Evaluating ATHB17Δ113 binding affinities with various mutations in targets DNA measured by OctectQK; locally fitted.**
(DOCX)

Table S2 **Phenology of** ***ATHB17*** **events and control.** The number of days to 50% silking and anthesis were measured and the number of days between anthesis and silking was calculated (ASI) each year for physiological studies conducted under standard agronomic practices conditions. Differences in phenology between *ATHB17* events and control were determined using a fixed effects model (as described in Materials and Methods), analyzing by year, by hybrid, by location.
(DOCX)

Table S3 **Dry matter accumulation in ATHB17 events and in control at R1 development stage.** Two independent *ATHB17* events in three genetic backgrounds were used in physiological studies conducted in 2011 and 2012 under standard agricultural practices (SAP). Data shown is for each hybrid in a given year by location using a fixed effects model (as described in Materials and Methods) to analyze dry matter accumulation data collected at R1. Ear partitioning coefficient was calculated by dividing ear dry weight by total dry weight and analyzed as described above. LSmean for the events and wild type control plants are shown in the table with respective delta, % delta and P-value.
(DOCX)
