# Supplementary figures and images for: Expression of a Truncated ATHB17 Protein in Maize Increases Ear Weight at Silking
Source: PLoS One. 2014 Apr 15;9(4):e94238. doi: 10.1371/journal.pone.0094238 (PMC3988052; doi:10.1371/journal.pone.0094238)

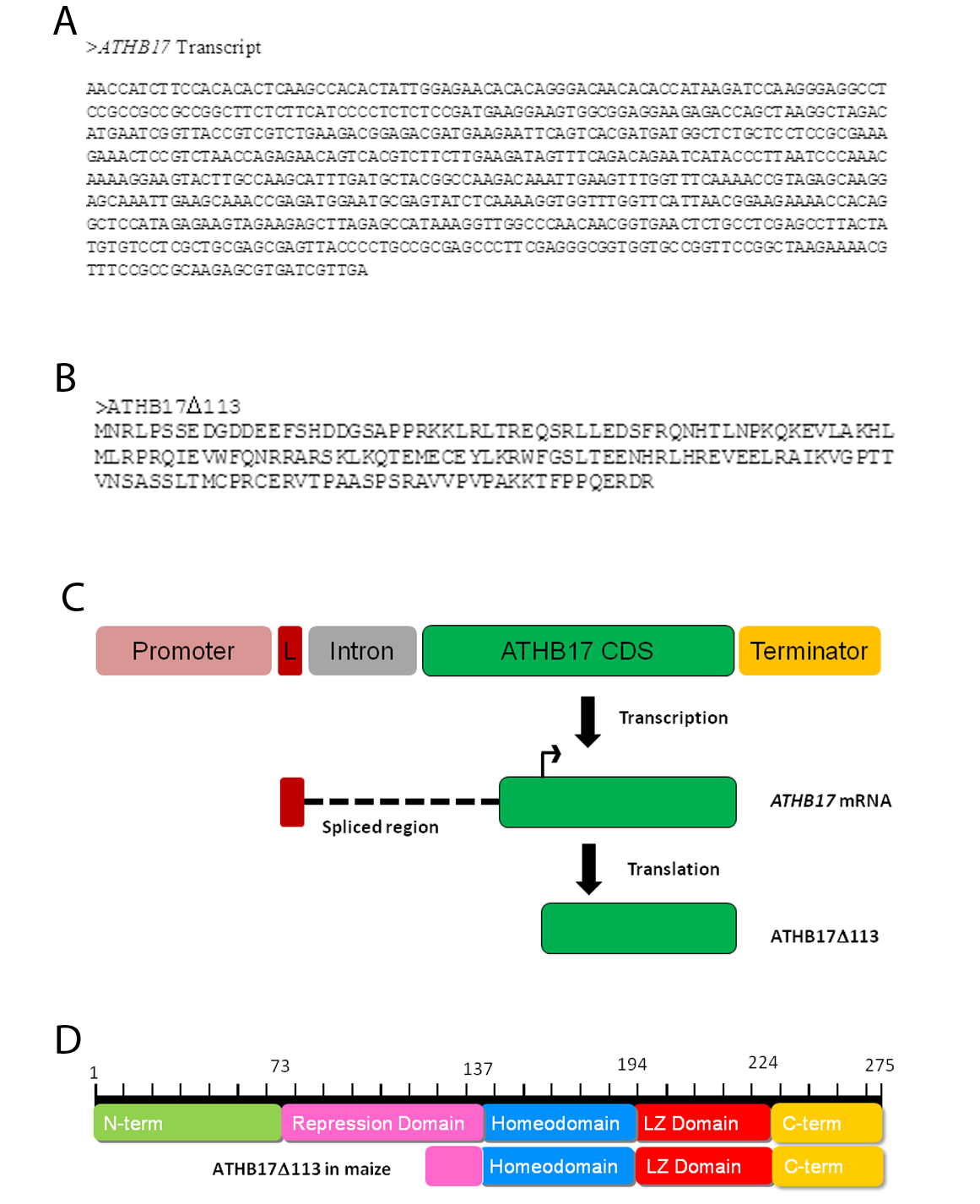

Supplement: Figure S1 — (A) ATHB17 Transcript sequence from transgenic event. (B) Predicted Amino Acid Sequence of the Protein Produced by ATHB17 RNA in transgenic event. The generated consensus sequence of ATHB17 transcript from the transgenic event was used in a BLASTX 2.2.23 search of the GenBank_Protein_Preferred database. The predicted amino acid sequence of ATHB17 protein starting with first methionine is shown. Compared to the Arabidopsis ATHB17 protein sequence (GenBank ID 179876107), the ATHB17 predicted protein sequence is truncated in transgenic event; lacking the amino-terminal 113 amino acids and is designated as ATHB17Δ113. (C) ATHB17 transcription and translation products in transgenic maize events. ATHB17 mRNA sequence identifies truncation of the 5′ region of the ATHB17 coding sequence. This transcript sequence is predicted to produce a truncated protein lacking the first 113 amino acids. (D) Domain structure of ATHB17. ATHB17 coding sequence contains canonical Homeodomain and Leucine Zipper (LZ) domains of HD-Zip family. Homeodomain is required for DNA binding. LZ domain is responsible for homodimerization and hetero-dimerrization with other HD-Zip II proteins. A repression domain is present upstream to the homeodomain. ATHB17 protein expressed in transgenic maize events lacks the first 113 amino acids resulting in deletion of part of repression domain containing EAR-like motif. (TIF) [file pone.0094238.s001.tif]

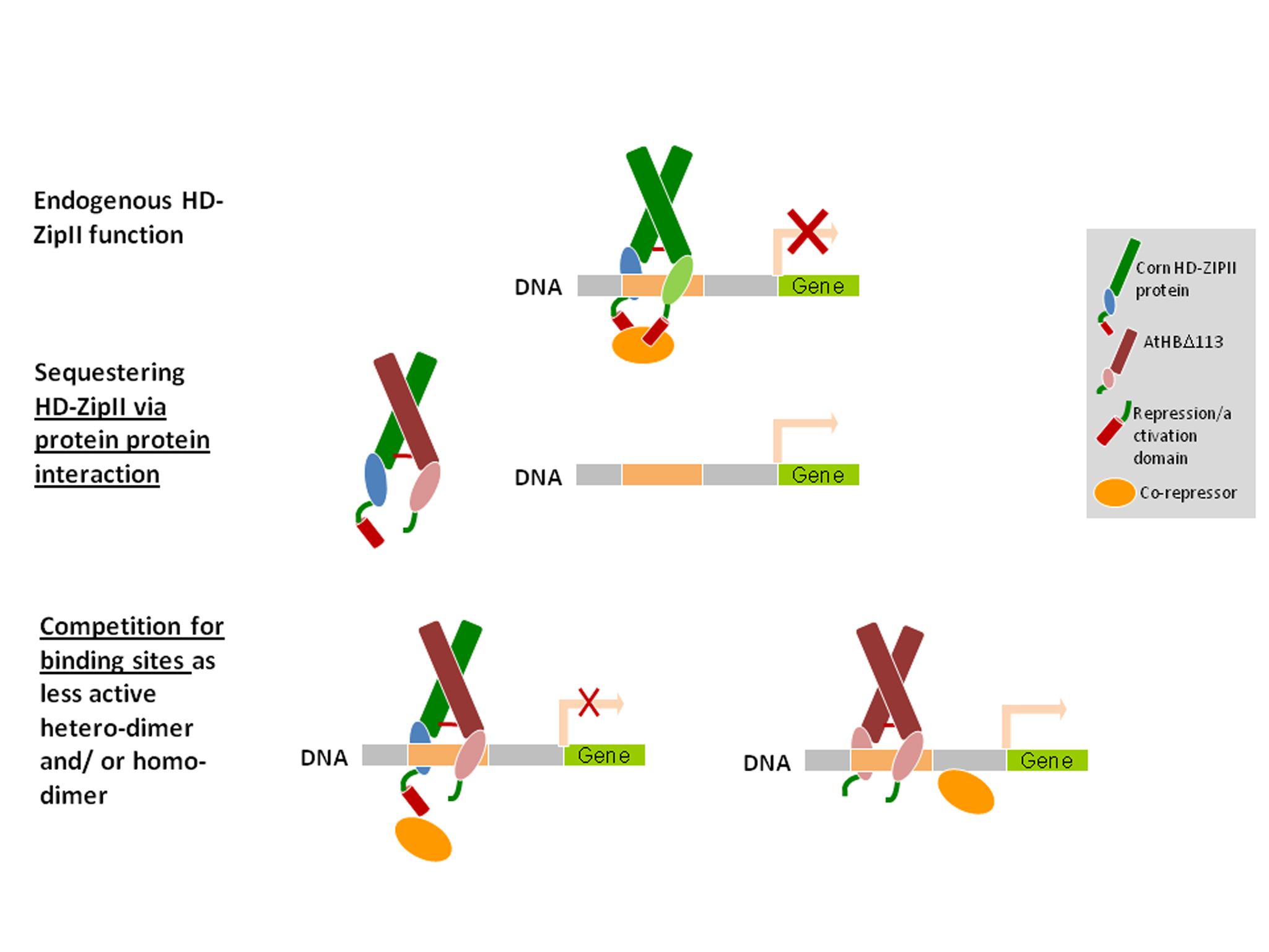

Supplement: Figure S2 — ATHB17Δ113 will function via dominant negative mechanism. ATHB17 is expressed as truncated protein in maize lacking part of the repression domain. Maize endogenous HD-Zip IIs function as transcriptional repressors. ATHB17Δ113 can interact with endogenous HD-Zip IIs and sequester endogenous proteins from binding to their targets resulting in relief of repression caused by maize HD-Zip IIs. In addition, heterodimer of ATHB17Δ113 and endogenous HD-Zip IIs or ATHB17Δ113 homodimers can compete for DNA binding resulting in altered target expression due to inability to cause active repression. (TIF) [file pone.0094238.s002.tif]
