# Supplementary material for: Expression of a Truncated ATHB17 Protein in Maize Increases Ear Weight at Silking
Source: PLoS One. 2014 Apr 15;9(4):e94238. doi: 10.1371/journal.pone.0094238 (PMC3988052; doi:10.1371/journal.pone.0094238)
Supplement: Table S1 — Evaluating ATHB17Δ113 binding affinities with various mutations in targets DNA measured by OctectQK; locally fitted. (DOCX) [file pone.0094238.s003.docx]

**Table S1:** **Evaluating ATHB17Δ113 binding affinities with various mutations in targets DNA measured by OctectQK; locally fitted.**

|  | **Sequences** | **K_D_**  **(nM)** | **Relative K*_D_***  **(Variant/Class II)** |
| --- | --- | --- | --- |
| **Class II** | CAGA**CAAT*C*ATTG**CGGC | 27.70 ±13 | 1.00 |
| **Class I** | CAGA**CAAT*T*ATTG**CGGC | 40.60 ±22 | 1.46 |
| **Non-consensus** | CAG**CTCAGTCTGA**CGGC | NB | -- |
| **Insertions in Class II pseudo-palindrome** | CAGA**CAAT*C*GATTG**CGGC | NB | -- |
|  | CAGA**CAATG*C*ATTG**CGGC­ | NB | -- |
|  | CAGA**CAATG*C*GATTG**CGGC | NB | -- |
|  | CAGA**CAAT*T*AATTG**CGGC | NB | -- |
|  | CAGA**CAATA*T*ATTG**CGGC | NB | -- |
|  | CAGA**CAAT*T*ACGAC**CGGC | NB | -- |
|  | CAGA**CAATGCCGAC**CGGC | NB | -- |
| **Substitution in Class II pseudo-palindrome** | CAGA**TAAT*C*ATTA**CGGC | 108 ±53 | 3.90 |
|  | CAGA**TAAT*T*ATTA**CGGC | 90.20 ±37 | 3.25 |
|  | CAGA**GAAT*C*ATTC**CGGC | NB | -- |
|  | CAGA**AAAT*C*ATTT**CGGC | 70.30 ±1 | 2.53 |
|  | CAGA**CTAT*C*ATAG**CGGC | NB | -- |
|  | CAGA**CGAT*C*ATCG**CGGC | NB | -- |
|  | CAGA**CCAT*C*ATGG**CGGC | NB | -- |
|  | CAGA**CATT*C*AATG**CGGC | NB | -- |
|  | CAGA**CAGT*C*ACTG**CGGC | NB | -- |
|  | CAGA**CACT*C*AGTG**CGGC | NB | -- |
|  | CAGA**CAAA*C*TTTG**CGGC | NB | -- |
|  | CAGA**CAAG*C*CTTG**CGGC | NB | -- |
|  | CAGA**CAAC*C*GTTG**CGGC | NB | -- |
| **Substitution in sequences flanking Class II pseudo-palindrome** | CACG**CAAT*C*ATTG**CGGC | 22.20 ±13 | 0.80 |
|  | CACG**CAAT*C*ATTG**GCGC | 39.80 ±38 | 1.43 |
|  | CAGT**CAAT*C*ATTG**TGGC | 28.10 ±22 | 1.01 |
|  | CAGA**CAAT*C*ATTG**AGGC | 27.60 ±22 | 0.99 |

Nucleotides highlighted in yellow correspond to mutational changes. Pseudo-palindrome (9-mer) is shown in bold. Nucleotides central to the pseudo-palindrome are in red (Class II) or in blue (Class I). NB indicates no binding.
