# Supplementary material for: Expression of a Truncated ATHB17 Protein in Maize Increases Ear Weight at Silking
Source: PLoS One. 2014 Apr 15;9(4):e94238. doi: 10.1371/journal.pone.0094238 (PMC3988052; doi:10.1371/journal.pone.0094238)
Supplement: Table S2 — Phenology of ATHB17 events and control. The number of days to 50% silking and anthesis were measured and the number of days between anthesis and silking was calculated (ASI) each year for physiological studies conducted under standard agronomic practices conditions. Differences in phenology between ATHB17 events and control were determined using a fixed effects model (as described in Materials and Methods), analyzing by year, by hybrid, by location. (DOCX) [file pone.0094238.s004.docx]

**Table S2.** **Phenology of *ATHB17* events and control.** The number of days to 50% silking and anthesis were measured and the number of days between anthesis and silking was calculated (ASI) each year for physiological studies conducted under standard agronomic practices conditions. Differences in phenology between *ATHB17* events and control were determined using a fixed effects model (as described in Materials and Methods), analyzing by year, by hybrid, by location.

| **Phase of Development** | **Year** | **Hybrid** | **Location** | **Event** | **Mean (days)** | **Control mean (days)** | **Delta (days)** | **% Delta** | **P-value** |
| --- | --- | --- | --- | --- | --- | --- | --- | --- | --- |
| Planting to 50% Silking | 2011 | NH6214 | ILW1 | Event 1 | 62.2 | 62.3 | -0.1 | -0.2 | 0.606 |
|  |  |  | ILW1 | Event 2 | 62.2 | 62.3 | -0.1 | -0.2 | 0.606 |
|  | 2011 | EXP257 | ILW1 | Event 1 | 61.9 | 62.0 | -0.1 | -0.2 | 0.549 |
|  |  |  | ILW1 | Event 2 | 61.8 | 62.0 | -0.2 | -0.3 | 0.335 |
|  |  |  | ILW2 | Event 1 | 52.7 | 53.0 | -0.3 | -0.6 | 0.360 |
|  |  |  | ILW2 | Event 2 | 52.9 | 53.0 | -0.1 | -0.1 | 0.818 |
|  |  |  | ILWR | Event 1 | 64.5 | 64.7 | -0.2 | -0.4 | 0.270 |
|  |  |  | ILWR | Event 2 | 64.5 | 64.7 | -0.2 | -0.4 | 0.273 |
|  | 2012 | NH6214 | ILW1 | Event 1 | 57.4 | 57.3 | 0.2 | 0.3 | 0.295 |
|  |  |  | ILW1 | Event 2 | 57.4 | 57.3 | 0.1 | 0.2 | 0.485 |
|  |  |  | ILW2 | Event 1 | 55.4 | 55.7 | -0.2 | -0.4 | 0.431 |
|  |  |  | ILW2 | Event 2 | 55.3 | 55.7 | -0.3 | -0.6 | 0.283 |
|  | 2012 | NN6306 | ILW1 | Event 1 | 57.4 | 57.3 | 0.1 | 0.2 | 0.549 |
|  |  |  | ILW1 | Event 2 | 57.3 | 57.3 | -0.1 | -0.1 | 0.665 |
|  |  |  | ILW2 | Event 1 | 55.2 | 55.3 | -0.2 | -0.3 | 0.538 |
|  |  |  | ILW2 | Event 2 | 54.9 | 55.3 | -0.4 | -0.7 | 0.183 |
|  | 2011-12 | Across Hybrids | Across loc | Event 1 | 58.4 | 58.5 | -0.1 | -0.2 | 0.299 |
|  |  |  | Across loc | Event 2 | 58.4 | 58.5 | -0.2 | -0.3 | 0.067 |
|  |  |  |  |  |  |  |  |  |  |
| **Phase of Development** | **Year** | **Hybrid** | **Location** | **Event** | **Mean (days)** | **Control mean (days)** | **Delta (days)** | **% Delta** | **P-value** |
| Planting to 50% Anthesis | 2011 | NH6214 | ILW1 | Event 1 | 62.3 | 62.5 | -0.2 | -0.3 | 0.367 |
|  |  |  | ILW1 | Event 2 | 62.6 | 62.5 | 0.1 | 0.2 | 0.649 |
|  | 2011 | EXP257 | ILW1 | Event 1 | 61.0 | 61.3 | -0.3 | -0.4 | 0.248 |
|  |  |  | ILW1 | Event 1 | 61.1 | 61.3 | -0.2 | -0.3 | 0.371 |
|  |  |  | ILW2 | Event 1 | 52.1 | 52.1 | 0.0 | 0.0 | 1.000 |
|  |  |  | ILW2 | Event 2 | 52.4 | 52.1 | 0.3 | 0.5 | 0.189 |
|  |  |  | ILWR | Event 1 | 64.0 | 64.4 | -0.4 | -0.6 | 0.224 |
|  |  |  | ILWR | Event 2 | 64.4 | 64.4 | 0.0 | 0.0 | 0.976 |
|  | 2012 | NH6214 | ILW1 | Event 1 | 57.3 | 57.2 | 0.2 | 0.3 | 0.283 |
|  |  |  | ILW1 | Event 2 | 57.3 | 57.2 | 0.1 | 0.2 | 0.474 |
|  |  |  | ILW2 | Event 1 | 55.3 | 55.4 | -0.1 | -0.2 | 0.580 |
|  |  |  | ILW2 | Event 2 | 55.2 | 55.4 | -0.2 | -0.3 | 0.469 |
|  | 2012 | NN6306 | ILW1 | Event 1 | 57.7 | 57.8 | -0.1 | -0.1 | 0.720 |
|  |  |  | ILW1 | Event 2 | 57.7 | 57.8 | -0.1 | -0.1 | 0.639 |
|  |  |  | ILW2 | Event 1 | 55.3 | 55.8 | -0.5 | -0.9 | 0.018 |
|  |  |  | ILW2 | Event 2 | 55.6 | 55.8 | -0.3 | -0.5 | 0.177 |
|  | 2011-12 | Across Hybrids | Across loc | Event 1 | 58.1 | 58.3 | -0.2 | -0.3 | 0.080 |
|  |  |  | Across loc | Event 2 | 58.2 | 58.3 | -0.1 | -0.1 | 0.548 |

|  |  |  |  |  |  |  |  |  |  |
| --- | --- | --- | --- | --- | --- | --- | --- | --- | --- |
| **Phase of Development** | **Year** | **Hybrid** | **Location** | **Event** | **Mean (days)** | **Control mean (days)** | **Delta (days)** | **% Delta** | **P-value** |
| Anthesis Silking Interval | 2011 | NH6214 | ILW1 | Event 1 | -0.10 | -0.20 | 0.10 | 50.0 | 0.604 |
|  |  |  | ILW1 | Event 2 | -0.40 | -0.20 | -0.20 | -100 | 0.303 |
|  | 2011 | EXP257 | ILW1 | Event 1 | 0.89 | 0.74 | 0.15 | 21.0 | 0.548 |
|  |  |  | ILW1 | Event 2 | 0.75 | 0.74 | 0.02 | 2.3 | 0.948 |
|  |  |  | ILW2 | Event 1 | 0.60 | 0.90 | -0.30 | -33.3 | 0.248 |
|  |  |  | ILW2 | Event 2 | 0.57 | 0.90 | -0.33 | -36.5 | 0.223 |
|  |  |  | ILWR | Event 1 | 0.46 | 0.34 | 0.12 | 34.5 | 0.457 |
|  |  |  | ILWR | Event 2 | 0.10 | 0.34 | -0.24 | -70.0 | 0.141 |
|  | 2012 | NH6214 | ILW1 | Event 1 | 0.11 | 0.11 | 0.00 | 0.0 | 1.000 |
|  |  |  | ILW1 | Event 2 | 0.11 | 0.11 | 0.00 | 0.0 | 1.000 |
|  |  |  | ILW2 | Event 1 | 0.18 | 0.29 | -0.12 | -40.0 | 0.611 |
|  |  |  | ILW2 | Event 2 | 0.11 | 0.29 | -0.18 | -62.2 | 0.423 |
|  | 2012 | NN6306 | ILW1 | Event 1 | -0.28 | -0.41 | 0.14 | 32.8 | 0.458 |
|  |  |  | ILW1 | Event 2 | -0.41 | -0.41 | 0.00 | 0.2 | 0.997 |
|  |  |  | ILW2 | Event 1 | -0.18 | -0.33 | 0.16 | 47.0 | 0.492 |
|  |  |  | ILW2 | Event 2 | -0.61 | -0.33 | -0.28 | -83.3 | 0.217 |
|  | 2011-12 | Across Hybrids | Across loc | Event 1 | 0.26 | 0.25 | 0.02 | 6.1 | 0.865 |
|  |  |  | Across loc | Event 2 | 0.09 | 0.25 | -0.16 | -63.9 | 0.075 |
