# Supplementary material for: Expression of a Truncated ATHB17 Protein in Maize Increases Ear Weight at Silking
Source: PLoS One. 2014 Apr 15;9(4):e94238. doi: 10.1371/journal.pone.0094238 (PMC3988052; doi:10.1371/journal.pone.0094238)
Supplement: Table S3 — Dry matter accumulation in ATHB17 events and in control at R1 development stage. Two independent ATHB17 events in three genetic backgrounds were used in physiological studies conducted in 2011 and 2012 under standard agricultural practices (SAP). Data shown is for each hybrid in a given year by location using a fixed effects model (as described in Materials and Methods) to analyze dry matter accumulation data collected at R1. Ear partitioning coefficient was calculated by dividing ear dry weight by total dry weight and analyzed as described above. LSmean for the events and wild type control plants are shown in the table with respective delta, % delta and P-value. (DOCX) [file pone.0094238.s005.docx]

**Table S3.** **Dry matter accumulation in ATHB17 events and in control at R1 development stage.** Two independent *ATHB17* events in three genetic backgrounds were used in physiological studies conducted in 2011 and 2012 under standard agricultural practices (SAP). Data shown is for each hybrid in a given year by location using a fixed effects model (as described in Materials and Methods) to analyze dry matter accumulation data collected at R1. Ear partitioning coefficient was calculated by dividing ear dry weight by total dry weight and analyzed as described above. LSmean for the events and wild type control plants are shown in the table with respective delta, % delta and P-value.

| **Trait Name** | **Year** | **Hybrid** | **Location** | **Event** | **Mean** | **Control mean** | **Delta** | **% Delta** | **P-value** |
| --- | --- | --- | --- | --- | --- | --- | --- | --- | --- |
| Ear dry weight (g/m^2^) | 2011 | NH6214 | ILW1 | Event 1 | 164.2 | 157.2 | 7.0 | 4.5 | 0.302 |
|  |  |  | ILW1 | Event 2 | 163.4 | 157.2 | 6.2 | 4.0 | 0.350 |
|  | 2011 | EXP257 | ILW1 | Event 1 | 178.9 | 166.4 | 12.5 | 7.5 | 0.271 |
|  |  |  | ILW1 | Event 2 | 169.7 | 166.4 | 3.3 | 2.0 | 0.773 |
|  |  |  | ILW2 | Event 1 | 85.1 | 78.6 | 6.5 | 8.3 | 0.381 |
|  |  |  | ILW2 | Event 2 | 93.7 | 78.6 | 15.1 | 19.2 | 0.046 |
|  |  |  | ILWR | Event 1 | 124.8 | 104.7 | 20.1 | 19.2 | 0.012 |
|  |  |  | ILWR | Event 2 | 106.6 | 104.7 | 1.9 | 1.8 | 0.795 |
|  | 2012 | NH6214 | ILW1 | Event 1 | 69.9 | 74.5 | -4.6 | -6.2 | 0.285 |
|  |  |  | ILW1 | Event 2 | 73.3 | 74.5 | -1.3 | -1.7 | 0.766 |
|  |  |  | ILW2 | Event 1 | 52.7 | 49.7 | 3.0 | 6.0 | 0.521 |
|  |  |  | ILW2 | Event 2 | 59.5 | 49.7 | 9.8 | 19.8 | 0.035 |
|  | 2012 | NN6306 | ILW1 | Event 1 | 61.7 | 54.7 | 6.9 | 12.7 | 0.108 |
|  |  |  | ILW1 | Event 2 | 65.5 | 54.7 | 10.7 | 19.6 | 0.014 |
|  |  |  | ILW2 | Event 1 | 55.0 | 46.1 | 8.9 | 19.3 | 0.056 |
|  |  |  | ILW2 | Event 2 | 52.0 | 46.1 | 5.9 | 12.8 | 0.194 |
|  | 2011-12 | Across Hybrids | Across loc | Event 1 | 99.2 | 93.1 | 6.2 | 6.6 | 0.006 |
|  |  |  | Across loc | Event 2 | 99.7 | 93.1 | 6.6 | 7.1 | 0.003 |

|  |  |  |  |  |  |  |  |  |  |
| --- | --- | --- | --- | --- | --- | --- | --- | --- | --- |
| **Trait Name** | **Year** | **Hybrid** | **Location** | **Event** | **Mean** | **Control mean** | **Delta** | **% Delta** | **P-value** |
| Stover dry weight (g/m^2^) | 2011 | NH6214 | ILW1 | Event 1 | 1142 | 1090 | 51.8 | 4.7 | 0.246 |
|  |  |  | ILW1 | Event 2 | 1103 | 1090 | 12.5 | 1.1 | 0.782 |
|  | 2011 | EXP257 | ILW1 | Event 1 | 962.2 | 899.8 | 62.5 | 6.9 | 0.254 |
|  |  |  | ILW1 | Event 2 | 957.2 | 899.8 | 57.4 | 6.4 | 0.320 |
|  |  |  | ILW2 | Event 1 | 991.8 | 955.9 | 35.9 | 3.8 | 0.191 |
|  |  |  | ILW2 | Event 2 | 963.8 | 955.9 | 7.9 | 0.8 | 0.761 |
|  |  |  | ILWR | Event 1 | 961.1 | 931.3 | 29.9 | 3.2 | 0.466 |
|  |  |  | ILWR | Event 2 | 893.5 | 931.3 | -37.8 | -4.1 | 0.358 |
|  | 2012 | NH6214 | ILW1 | Event 1 | 1004 | 976.4 | 27.3 | 2.8 | 0.257 |
|  |  |  | ILW1 | Event 2 | 1005 | 976.4 | 28.3 | 2.9 | 0.240 |
|  |  |  | ILW2 | Event 1 | 958.5 | 921.6 | 36.9 | 4.0 | 0.192 |
|  |  |  | ILW2 | Event 2 | 979.4 | 921.6 | 57.8 | 6.3 | 0.039 |
|  | 2012 | NN6306 | ILW1 | Event 1 | 1019 | 998.2 | 20.7 | 2.1 | 0.390 |
|  |  |  | ILW1 | Event 2 | 996.7 | 998.2 | -1.5 | -0.2 | 0.950 |
|  |  |  | ILW2 | Event 1 | 954.5 | 904.3 | 50.2 | 5.6 | 0.073 |
|  |  |  | ILW2 | Event 2 | 909.7 | 904.3 | 5.4 | 0.6 | 0.843 |
|  | 2011-12 | Across Hybrids | Across loc | Event 1 | 993.2 | 956.6 | 36.6 | 3.8 | 0.114 |
|  |  |  | Across loc | Event 2 | 951.4 | 956.6 | -5.1 | -0.5 | 0.804 |

|  |  |  |  |  |  |  |  |  |  |
| --- | --- | --- | --- | --- | --- | --- | --- | --- | --- |
| **Trait Name** | **Year** | **Hybrid** | **Location** | **Event** | **Mean** | **Control mean** | **Delta** | **% Delta** | **P-value** |
| Total dry weight (g/m^2^) | 2011 | NH6214 | ILW1 | Event 1 | 1274 | 1280 | -5.4 | -0.4 | 0.903 |
|  |  |  | ILW1 | Event 2 | 1311 | 1280 | 31.3 | 2.4 | 0.469 |
|  | 2011 | EXP257 | ILW1 | Event 1 | 1066 | 1114 | -48.5 | -4.3 | 0.438 |
|  |  |  | ILW1 | Event 2 | 1174 | 1114 | 59.5 | 5.3 | 0.328 |
|  |  |  | ILW2 | Event 1 | 1077 | 1035 | 42.5 | 4.1 | 0.187 |
|  |  |  | ILW2 | Event 2 | 1057 | 1035 | 23.0 | 2.2 | 0.454 |
|  |  |  | ILWR | Event 1 | 1086 | 1036 | 49.9 | 4.8 | 0.274 |
|  |  |  | ILWR | Event 2 | 1000 | 1036 | -35.9 | -3.5 | 0.427 |
|  | 2012 | NH6214 | ILW1 | Event 1 | 1074 | 1051 | 22.8 | 2.2 | 0.371 |
|  |  |  | ILW1 | Event 2 | 1078 | 1051 | 27.0 | 2.6 | 0.289 |
|  |  |  | ILW2 | Event 1 | 1011 | 971.2 | 39.8 | 4.1 | 0.205 |
|  |  |  | ILW2 | Event 2 | 1039 | 971.2 | 67.4 | 6.9 | 0.031 |
|  | 2012 | NN6306 | ILW1 | Event 1 | 1081 | 1053 | 27.7 | 2.6 | 0.278 |
|  |  |  | ILW1 | Event 2 | 1062 | 1053 | 9.2 | 0.9 | 0.718 |
|  |  |  | ILW2 | Event 1 | 1009 | 950.1 | 59.2 | 6.2 | 0.057 |
|  |  |  | ILW2 | Event 2 | 961.5 | 950.1 | 11.3 | 1.2 | 0.709 |
|  | 2011-12 | Across Hybrids | Across loc | Event 1 | 1069 | 1052 | 16.8 | 1.6 | 0.482 |
|  |  |  | Across loc | Event 2 | 1055 | 1052 | 3.1 | 0.3 | 0.892 |

|  |  |  |  |  |  |  |  |  |  |
| --- | --- | --- | --- | --- | --- | --- | --- | --- | --- |
| **Trait Name** | **Year** | **Hybrid** | **Location** | **Event** | **Mean** | **Control mean** | **Delta** | **% Delta** | **P-value** |
| Ear Partitioning Coefficient | 2011 | NH6214 | ILW1 | Event 1 | 0.299 | 0.296 | 0.003 | 1.1 | 0.349 |
|  |  |  | ILW1 | Event 2 | 0.301 | 0.296 | 0.005 | 1.7 | 0.137 |
|  | 2011 | EXP257 | ILW1 | Event 1 | 0.153 | 0.156 | -0.003 | -1.9 | 0.568 |
|  |  |  | ILW1 | Event 2 | 0.152 | 0.156 | -0.004 | -2.3 | 0.507 |
|  |  |  | ILW2 | Event 1 | 0.079 | 0.076 | 0.003 | 4.5 | 0.545 |
|  |  |  | ILW2 | Event 2 | 0.088 | 0.076 | 0.013 | 16.8 | 0.028 |
|  |  |  | ILWR | Event 1 | 0.115 | 0.101 | 0.014 | 13.7 | 0.018 |
|  |  |  | ILWR | Event 2 | 0.107 | 0.101 | 0.006 | 5.5 | 0.315 |
|  | 2012 | NH6214 | ILW1 | Event 1 | 0.068 | 0.074 | -0.006 | -7.6 | 0.169 |
|  |  |  | ILW1 | Event 2 | 0.071 | 0.074 | -0.003 | -4.2 | 0.442 |
|  |  |  | ILW2 | Event 1 | 0.051 | 0.050 | 0.001 | 1.1 | 0.888 |
|  |  |  | ILW2 | Event 2 | 0.057 | 0.050 | 0.006 | 12.7 | 0.110 |
|  | 2012 | NN6306 | ILW1 | Event 1 | 0.060 | 0.053 | 0.007 | 13.1 | 0.090 |
|  |  |  | ILW1 | Event 2 | 0.064 | 0.053 | 0.011 | 21.5 | 0.006 |
|  |  |  | ILW2 | Event 1 | 0.054 | 0.047 | 0.006 | 13.6 | 0.110 |
|  |  |  | ILW2 | Event 2 | 0.053 | 0.047 | 0.006 | 13.3 | 0.112 |
|  | 2011-12 | Across Hybrids | Across loc | Event 1 | 0.089 | 0.086 | 0.003 | 3.9 | 0.085 |
|  |  |  | Across loc | Event 2 | 0.091 | 0.086 | 0.005 | 5.6 | 0.013 |
